# Mandibulate convergence in an armoured Cambrian stem chelicerate

**DOI:** 10.1186/s12862-017-1088-7

**Published:** 2017-12-21

**Authors:** Cédric Aria, Jean-Bernard Caron

**Affiliations:** 1 Department of Ecology and Evolutionary Biology, University of Toronto, Toronto, ON M5S3B2 Canada; 2 Department of Natural History (Palaeobiology Section), Royal Ontario Museum, Toronto, ON M5S2C6 Canada; 3 Department of Earth Sciences, University of Toronto, Toronto, ON M5S3B1 Canada; 4 Present address: State Key Laboratory of Palaeobiology and Stratigraphy, Nanjing Institute of Geology and Palaeontology, Chinese Academy of Sciences, Nanjing, 210008 China

**Keywords:** Arthropoda, Chelicerata, Convergence, Macroevolution, Cambrian, Burgess Shale

## Abstract

**Background:**

Chelicerata represents a vast clade of mostly predatory arthropods united by a distinctive body plan throughout the Phanerozoic. Their origins, however, with respect to both their ancestral morphological features and their related ecologies, are still poorly understood. In particular, it remains unclear whether their major diagnostic characters were acquired early on, and their anatomical organization rapidly constrained, or if they emerged from a stem lineage encompassing an array of structural variations, based on a more labile “panchelicerate” body plan.

**Results:**

In this study, we reinvestigated the problematic middle Cambrian arthropod *Habelia optata* Walcott from the Burgess Shale, and found that it was a close relative of *Sanctacaris uncata* Briggs and Collins (in Habeliida, ord. nov.), both retrieved in our Bayesian phylogeny as stem chelicerates. *Habelia* possesses an exoskeleton covered in numerous spines and a bipartite telson as long as the rest of the body. Segments are arranged into three tagmata. The prosoma includes a reduced appendage possibly precursor to the chelicera, raptorial endopods connected to five pairs of outstandingly large and overlapping gnathobasic basipods, antennule-like exopods seemingly dissociated from the main limb axis, and, posteriorly, a pair of appendages morphologically similar to thoracic ones. While the head configuration of habeliidans anchors a seven-segmented prosoma as the chelicerate ground pattern, the peculiar size and arrangement of gnathobases and the presence of sensory/tactile appendages also point to an early convergence with the masticatory head of mandibulates.

**Conclusions:**

Although habeliidans illustrate the early appearance of some diagnostic chelicerate features in the evolution of euarthropods, the unique convergence of their cephalons with mandibulate anatomies suggests that these traits retained an unusual variability in these taxa. The common involvement of strong gnathal appendages across non-megacheirans Cambrian taxa also illustrates that the specialization of the head as the dedicated food-processing tagma was critical to the emergence of both lineages of extant euarthropods—Chelicerata and Mandibulata—and implies that this diversification was facilitated by the expansion of durophagous niches.

**Electronic supplementary material:**

The online version of this article (10.1186/s12862-017-1088-7) contains supplementary material, which is available to authorized users.

## Background

The early evolution of arthropods has long been emblematic of the “Cambrian Explosion” [1, 2], and has grown through the past decades into a remarkable variety of “stem” taxa [3–9], attracting emphasis on phylogenetic similarities (homologies) and exceptional divergences between morphologies. This is because morphological similarities of a common descent allow for the placement of “weird wonders” on a phylogenetic tree [3, 5, 10, 11], while the emphasis on divergences promotes the idea of unusual morphological variability in the early evolution of clades [2]. Homoplasies, by contrast, have received less attention in that context [12].

In spite of being discarded when reconstructing phylogenetic relationships, phenotypic convergence remains an informative (palaeo)biological observation, as it can also illustrate the formidable morphological variability deployed within or across body plans in response to similar selective pressures [13–16]. In the Cambrian, remarkable cases of convergence have been documented in which body plans or appendages are reminiscent of derived taxa due to the early occupation of the same niches [17, 18]. At the same time, convergences are well known to outline some of the structural constraints shared by different groups of species. A prominent case of convergence involving all types of Palaeozoic and extant euarthropods is the distal differentiation of appendages into pincers [9, 12].

Broad morpho-functional convergences may be less expected between early-diverging lineages, as we would think that recently selected traits would provide greater fitness for the exploration of new niches, and that disruptive selection would impact the phenotype at large—even when considering the role of mosaic evolution. Thus, for instance, early representatives of the two extant euarthropod clades, Chelicerata and Mandibulata, would have been morphologically diverging rather than converging, developing different morpho-functional adaptations and “maturing” their respective body plans to form the basis of their great diversification during the Phanerozoic.

However, little is still known about the origin per se of extant lineages and about the significance of Cambrian high evolutionary rates [19] and, questionably [20, 21], exceptional morphological variability [2, 22, 23] on their stem taxa. We recently proposed a scenario for the early radiation of Mandibulata based on a reevaluation of bivalved arthropod anatomy [9], which represents a new avenue of research based on adult macrofossils to explore these questions. The origin of chelicerates, on the other hand, has been the subject of some more active debate in recent years [24–28]. Based on the topology and structural similarity between chelicerae and “great appendages,” some authors [24, 25, 29] have proposed a phylogenetic continuity between these frontal limbs. Although this does not imply that megacheirans (bearing the great appendages) and chelicerates necessarily form a clade, this has been hypothesized as such [12, 28, 30, 31], assuming that the chelate nature of these appendages constitutes an apomorphy for these taxa, contrasting with the antennular anteriormost appendages present in other euarthropods.

Although the grouping of megacheirans with chelicerates has been retrieved phylogenetically [6], it has been shown to be a possible methodological (polarization) bias associated with the retrieval of Arachnomorpha under parsimony [27]. More recent analyses have found megacheirans (or, at least, cheiromorphs) to be more basal, possibly forming a sister group to artiopodans and extant clades [8, 9]. Apart from considering great appendages to be the direct precursors of the chelicerae, there does not seem indeed to be any unambiguous character supporting the placement of megacheirans on the chelicerate lineage.

Instead of megacheirans, the groundplan of chelicerates could be represented by the Burgess Shale species *Sanctacaris* [26], as originally proposed by Briggs and Collins [32]. *Sanctacaris* has been shown so far to display a five- or six-segmented cephalon, a condition closer to the euchelicerate condition (six-segmented) than the four-segmented head of megacheirans. However—and there lies the conundrum—, in addition to several uncertainties regarding its head configuration, such as the forward attachment of its raptorial limbs in a “bundle,” *Sanctacaris* lacks a clear indication of a chelate frontalmost appendage comparable to the chelicera. A series of very recent studies have documented other forms with likely affinities to *Sanctacaris* from the Spence and Wheeler Shales in Utah [33, 34] and the Emu Bay Shale in Australia [35], but this particular uncertainty still remains.

It may also be considered that both megacheirans (or at least some of them) and *Sanctacaris*-like morphotypes are part of the chelicerate lineage, with *Sanctacaris* bearing, for instance, very reduced great appendages. This idea would conflict with a scenario in which antennule-bearing artiopodans (and in particular xenopodans, i.e., *Sidneyia* and *Emeraldella*) would constitute the basalmost part of this lineage, based mainly on a comparison between their exopod morphology and the gill opercula of euchelicerates [26]. Such comparison remains tentative, however, and further assessment of possible chelicerate affinities are notably hampered by a limited knowledge of the xenopodan head anatomy.

In this study, we thoroughly reinvestigated the Burgess Shale euarthropod *Habelia* Walcott, 1912 based on Walcott’s original material and new specimens discovered by the Royal Ontario Museum. *Habelia optata* was initially regarded by Walcott as an “aglaspidid merostome,” which would hint at a chelicerate affinity [36], but this statement lacked much justification [37]. Simonetta [38] and Simonetta and Delle Cave [39] followed this view based mostly on overall aspect, while preferring to compare *H. brevicauda*, the new morphotype erected by Simonetta, to *Leanchoilia* [39]—a megacheiran. Importantly, early authors [37–41] recognized the presence of at least five pairs of head appendages, a condition that could have later related this animal to *Sanctacaris*—even if an interpretation of strictly five pairs and some other morphological details led to comparisons with crustaceans instead [40, 42]. In his revision of the genus, however, Whittington [43] rejected previous interpretations of a cephalon with five head appendages or more, leaving *Habelia* as a *problematicum*.

Herafter, we reevaluate the significance of *Habelia* for the early evolution of chelicerates, as well as for the understanding of morphological convergence in the ecological context of the radiation of Cambrian euarthropods.

## Methods

### Fossil material and observation

Most specimens were collected in situ from the Greater Phyllopod Bed within the Burgess Shale Walcott Quarry, in Yoho National Park, British Columbia. Two additional specimens come from other Burgess Shale localities (see Additional file 1 for a detailed list of studied specimens). 41 specimens were studied in total, including 14 from the National Museum of Natural History, Washington D.C. (USNM), and 27 new specimens from the Royal Ontario Museum Invertebrate Palaeontology collections in Toronto (ROMIP). Methodology for observation followed our previous studies on Burgess Shale arthropods [9, 27, 44]. Selected specimens were mechanically prepared to remove matrix covering anatomical features. All material was observed under a stereomicroscope equipped with polarizing filters, and photographed both dry and wet under natural and cross-polarized lighting, occasionally using ammonium chloride sublimate to highlight three-dimensional details.

### Phylogenetic analyses

We used a Bayesian technique of tree search on a revised version of a previously published dataset [9], now comprising 77 taxa and 215 characters, all unweighted and unordered (see Additional file 2, ref. [9] for overall character descriptions and Additional file 1 for improvements), and with inapplicable entries treated as uncertainties. The topology was generated using MrBayes v.3.2.6 [45], with parameter settings following the Mkv method [46]. As per MrBayes’ restrictions, Priapulida was used as the single outgroup (following refs. [47, 48]), but was subsequently retrieved in a polytomy with Nematoda. Trees were produced during four runs of 5,000,000 generations with four parallel chains, a tree sampled every 1000 generation and burn-in of 20%. Among-character rates were set to remain equal. A Bayesian treatment of our data was chosen to explore an alternative methodology to parsimony, which is particularly sensitive to the suboptimal treatment of inapplicable states [27]. Bayesian analyses have also been shown to provide more accurate results than parsimony, albeit with a possible loss of precision [49, 50]. In order to account for the morphological and molecular signal that can only be coded among extant taxa, we chose to apply a backbone constraint on our dataset, based on the topology of Regier et al. [51]. As in ref. [9], we preferred this method over the direct implementation of numerous extant-only characters to avoid overburdening the dataset with question marks, which causes instability and a lack of resolution in (relatively) small datasets [52, 53]. Finding inconsistencies of placement with different codings of the head anatomy, pycnogonids were removed from the analysis presented here (see Phylogenetic results).

### Abbreviations and terminology

*Abbreviations used in figures*: ag, anterior gnathobase; am, arthrodial membrane; an, anus; ap, anal pouch; att, endopod attachment on gnathobase; bas, basipod(s); ce, cephalic endopod(s); ce*n*, cephalic endopod *n*; cel, left cephalic exopods; cpl, cephalic pleura; cx, cephalic exopod(s); cx*n*, cephalic exopod *n*; db, distal brush; dpex, distal part of exopod; ds, dorsal spine; dtp; distal telson piece; e, eye; e*n*, endopod *n*; en, endopod; ex, exopod; das, dark stain; g, gnathobase(s); g*n*l, left gnathobase *n*; g*n*r, right gnathobase *n*; hyp, hypostome; ia, intermediary appendage; it, intestine; jt, joint; la, labrum; m, mouth; nv?, nerve?; oe, oesophagus; p*n*, podomere *n*; pex, posterior exopod(s); pex*n*, posterior exopod *n*; ppex, proximal part of exopod; ptp, proximal telson piece; rap, reduced anterior appendage(s); st, stomach; t*n*, thoracic appendage *n*; te, thoracic endopod(s); tel., telson, te*n*, thoracic endopod *n*; th, telson head; tpl, trunk pleura(e). Hereafter, we call “gnathobasic appendage” an appendage whose basipod is gnathobasic, that is, differentiated into a masticatory structure (more strongly sclerotized, often bearing ornaments such as setae and teeth).

## Results

### Systematic palaeontology

Superphylum **Panarthropoda** Nielsen, 1995.

Phylum **Euarthropoda** Lankester, 1904.

Clade **Arachnomorpha** Heider, 1913 (= Arachnata Lauterbach, 1973).

**Diagnosis (emended from Størmer, 1944).** Euarthropods with the following characters: Cephalic shield encompassing at least four pairs of appendages with well-developed endopods; originally, presence along body of at least one pair of appendages with basipod differentiated into a well-sclerotized gnathal sclerite bearing setae or teeth (“gnathobasic appendage”); third gnathobasic cephalic appendage also part of groundplan; post-cephalic endopods terminating in a trident of claws with various arrangements.

Order **Habeliida**, **ord. nov. Aria and Caron**

**Type family**. Habeliidae Simonetta and Delle Cave, 1975.

**Other included taxa**. Sanctacarididae Legg and Pates, 2016.

**Diagnosis.** Arachnomorph arthropods with the following characters: Cephalic shield with sub-triangular, sub-horizontal pleural expansions and with antero-lateral notches accommodating pair of lateral compound eyes with no peduncle; cephalic shield with large mesio-dorsal bulge accommodating stomach; five pairs of anterior, slender and segmented antennule-like exopods likely inserted below the eyes and dorsally to other head appendages; on ventral side of head, reduced pair of appendages inserted anteriormost (presumed in Sanctacarididae), followed by five pairs of appendages composed of gnathobasic basipods increasing in size posteriad and bearing seven-segmented spinose/setose enditic endopods projecting anteriad; trunk bearing paddle-like exopods fringed with thin lamellae.

**Remarks.** We maintain the family Sanctacarididae erected by Legg and Pates [33], since 10 trunk segments and a spatulate telson remain diagnostic of *Sanctacaris uncata*, *Utahcaris orion* [33] and *Wisangocaris barbarahardyae* [35]. The affinity of *Messorocaris magna* [34] is less clear, but the peculiar shape of its trunk pleurae may place it in its own family.

*Habelia* had previously been assigned to the orders Aglaspina by Walcott and Emeraldellia by Størmer [36]. Given the lack of cladistic support for these taxa, which would be para- or polyphyletically nested within Arachnomorpha, the lack of redescription for *Molaria*, and the fact that their diagnoses should be extensively revised in light of the new data gathered on aglaspidids and *Emeraldella*, we have not reused Aglaspina or Emeraldellia herein.

Family **Habeliidae** Simonetta and Delle Cave, 1975.

**Type genus.** *Habelia* Walcott, 1912.

**Diagnosis.** Habeliidan euarthropods with the following characters: Body elongate, 19-segmented, divided into three distinct tagmata: cephalon (or “prosoma”) of seven segments (or eight somites) and trunk (12 segments) composed of a five-segmented thorax (or “mesosoma”) and eight-segmented post-thorax (or “metasoma”); trunk tagmatization based on discrete limb differentiation between thorax and post-thorax; posteriormost cephalic appendage (7th) similar to thoracic appendages, all characterized by a cheiromorph morphology: large undifferentiated basipods, well-developed seven-segmented endopods without endites, and paddle-like exopods fringed with oblanceolate lamellae; telson elongate.

**Remarks.** We hereby establish a diagnosis for the family Habeliidae, as the original publication of the taxon was not associated with one [39]; we also formalize diagnoses and descriptions for *Habelia optata* hereafter. The genus *Thelxiope* was also included in Habeliidae by Simonetta and Delle Cave; however, the presence of eight post-cephalic tergites and a pygidium would rather seem to indicate a relationship with *Mollisonia* [54, 55]. *Thelxiope* is therefore removed from Habeliidae.

**Fig. 1:**
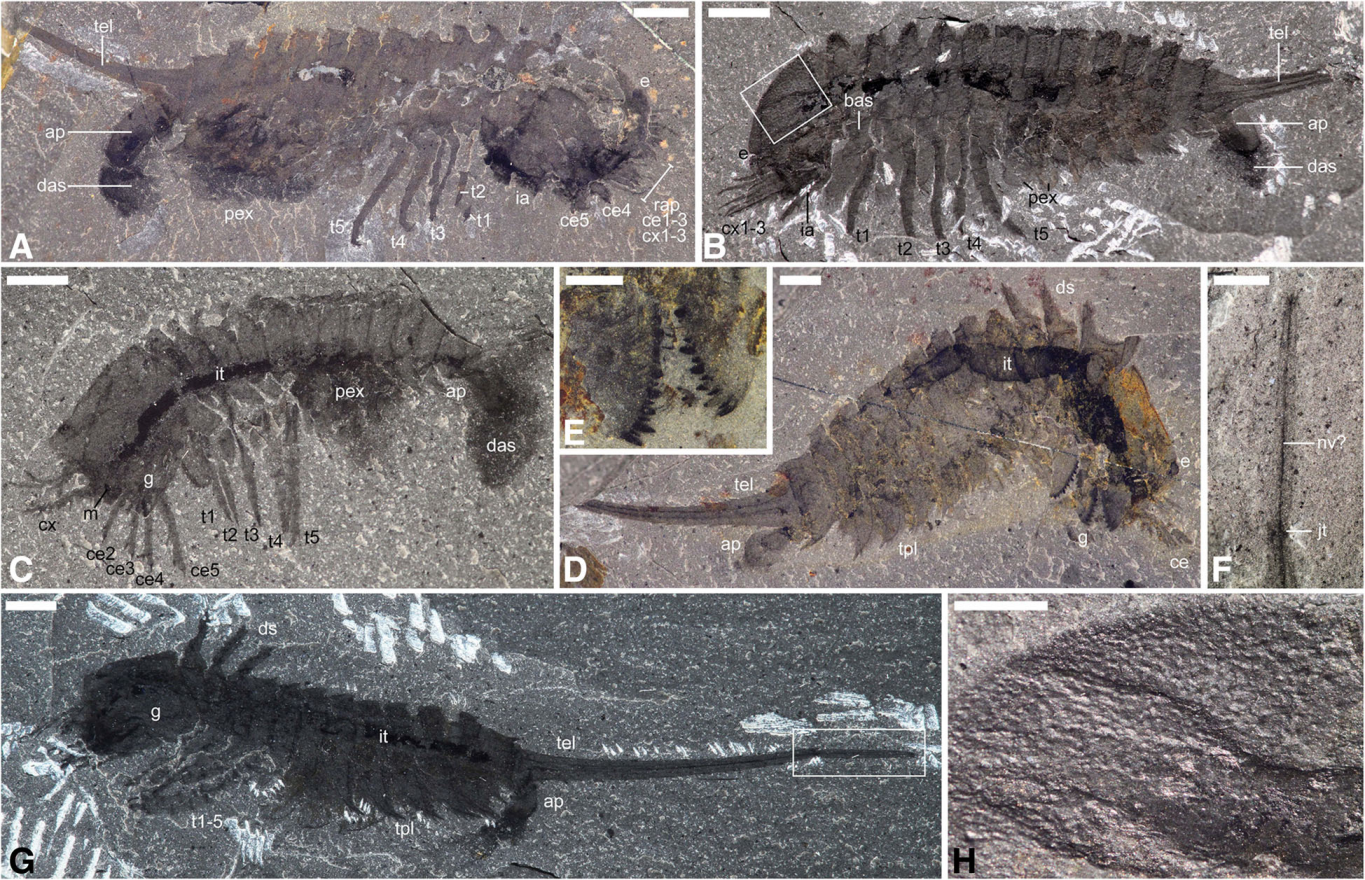
General anatomy of *Habelia optata*, morphs A (**d**-**g**) and B (**a**-**c**, **h**). **a** ROMIP 64357. **b** USNM 139209 (inset is (**h**)). **c** ROMIP 64358. **d** ROMIP 64359. **e** Close-up of the mandibles on the counterpart of (**d**) (wet specimen). **f** Close-up of the distal telson piece in (**g**) (wet specimen). **g** Holotype USNM 57693 (inset is (**f**)). **h** Close-up on cephalic ornamentation akin to trilobite prosopon in (**b**). All pictures taken under cross-polarized light. For abbreviations, see Methods. Scale bars: (**a**), 4 mm; (**b**), 3 mm; (**c**, **d**, **g**), 2 mm; (**e**, **f**, **h**), 1 mm

**Fig. 2:**
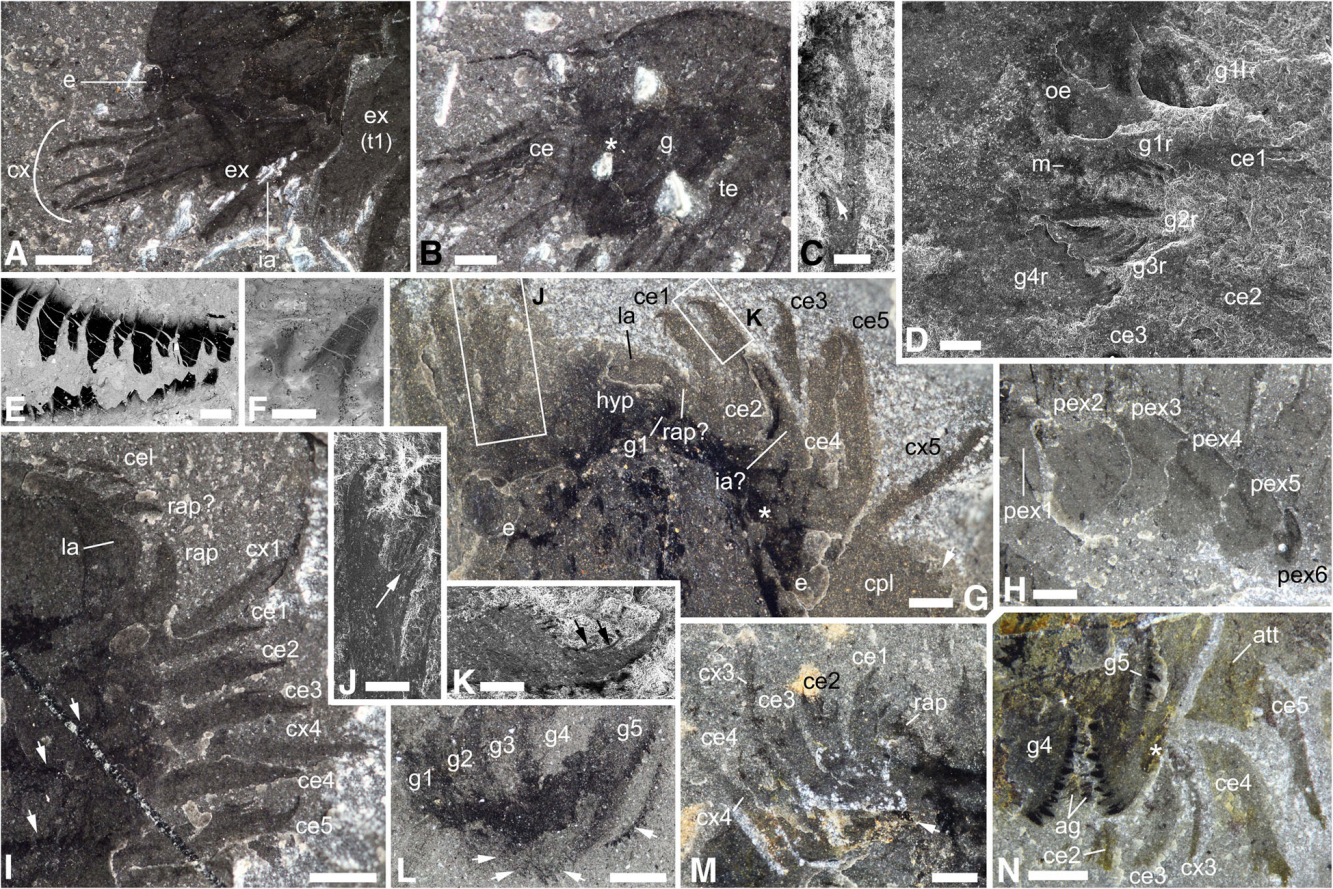
Anatomical and morphological details of *Habelia optata*, morphs A (**b**, **d**, **f**, **n**) and B (**a**, **e**, **g**, **i**, **l**, **m**). **a** USNM 139209, close-up of anterior cephalic area, showing intermediary appendage. **b** USNM 268931, cephalon, showing superimposed insertion of endopods on gnathobases; star points to insertion of anterior endopods. **c** ROMIP 64357, close-up of fourth cephalic exopodial branch, distal portion showing slender podomeres; arrow points to trident of setae at podomere junction. **d** ROMIP 64358, close-up of anteriormost region, showing mouth opening and first anterior pairs of gnathobases. **e** ROMIP 64360, close-up of teeth on masticatory margin of gnathobase; note heavy concentration of carbon in teeth. **f** Close-up of teeth on masticatory margin of posterior gnathobase on same specimen as in D, showing stronger carbon content in dental edge. **g** ROMIP 64364, specimen preserved in ventral aspect, close-up of anterior region showing labrum, eyes and appendages; star marks attachment of fifth spinose endopod; arrow points at ornamental spine of cephalic pleura; insets as indicated. **h** ROMIP 64362, close-up of posterior trunk exopods. **i** ROMIP 64363, close-up of anterior right cephalic region, dorsal view showing labrum and appendages; arrows point to overprint of gnathobases underneath cephalon. **j, k** ROMIP 64364. **j** Close-up of distal portion of cephalic endopod, showing “platform” with setal brushes. **k** Close-up of terminal claw; arrows point to teeth on inner margin of claw. **l** USNM 144907, close-up of cephalic gnathobases; arrows point to dentate margins of opposing gnathobases. **m** ROMIP 64357, close-up on anterior left cephalic region, showing appendages; arrow points to anterior insertion of fourth cephalic endopod. **n** ROMIP 64359, close-up of cephalic appendages showing insertion of endopods on gnathobases; star marks attachment of fourth cephalic endopod on its gnathobase. **c-f, j** and **k** are SEM images; all other are stereomicroscope images of dry specimens under cross-polarized lighting. For abbreviations, see Methods. Scale bars: (**a**, **g**, **h**, **i**, **l**, **n**), 1 mm; (**b**, **m**), 0.5 mm; (**c**, **d**, **k**), 200 μm; (**e**), 100 μm; (**f**), 50 μm; (**j**), 500 μm

**Fig. 3:**
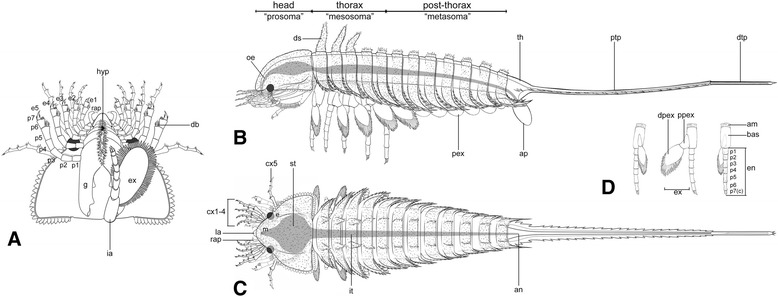
Diagrammatic reconstruction of *Habelia optata*, morph A. **a** Ventral view of the cephalon. Right “intermediary” appendage removed to show gnathobase morphology. **b** Lateral view. **c** Dorsal view. **d** Isolated biramous thoracic limb in frontal, lateral and posterior views (left to right). For abbreviations, see Methods. Line drawings courtesy of Joanna Liang © Royal Ontario Museum

**Fig. 4:**
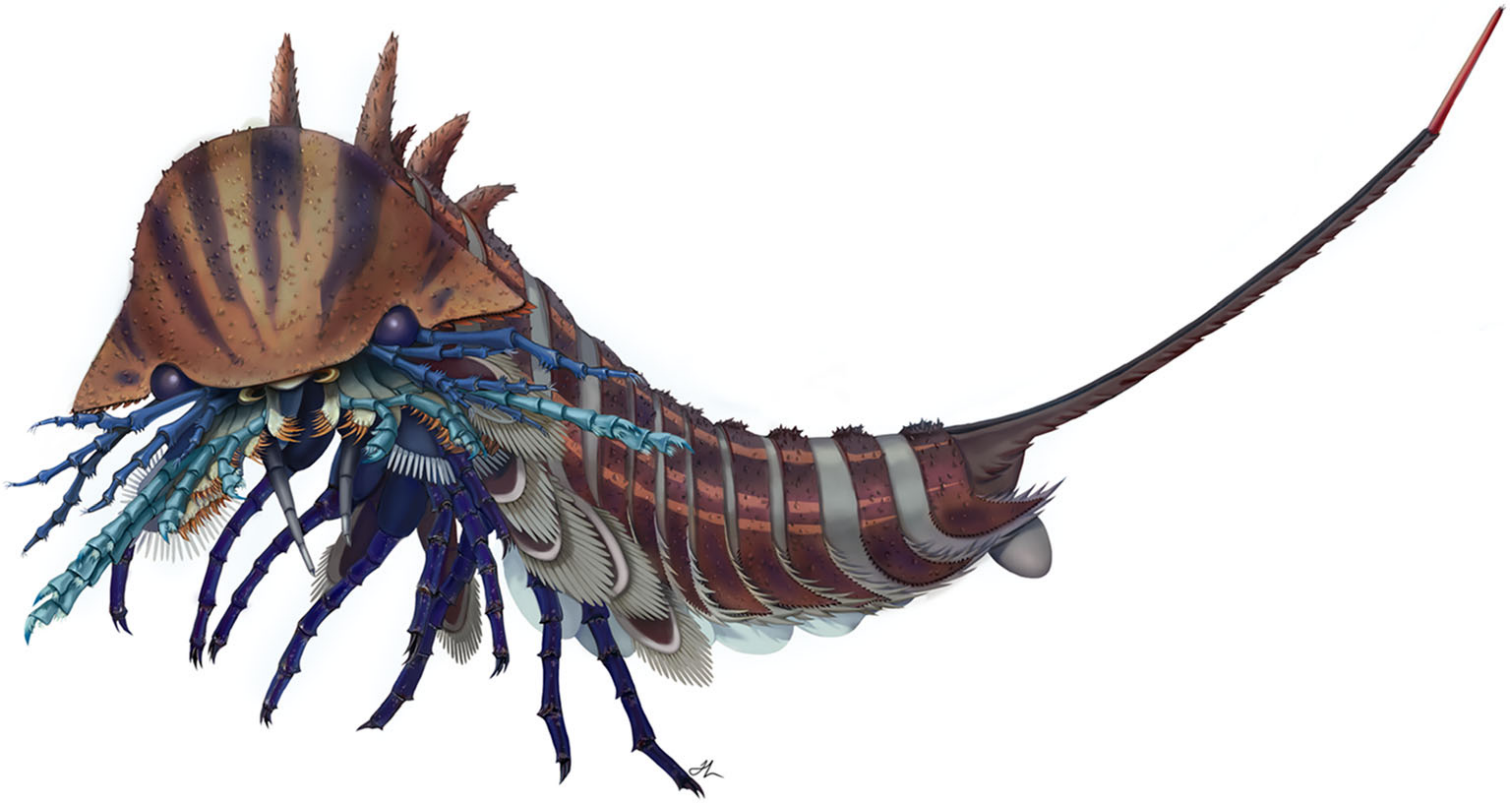
Artistic reconstruction of *Habelia optata*. Courtesy of Joanna Liang © Royal Ontario Museum

Genus ***Habelia*** Walcott, 1912.

(Figures 1, 2, 3, 4; Additional file 3, Additional file 4, Additional file 5, Additional file 6, Additional file 7 and Additional file 8)

**Type species.** *Habelia optata* Walcott, 1912.

**Diagnosis.** Habeliid arthropod with the following characters: Post-ocular lateral and postero-lateral cephalic margins as well as pleural margins of trunk segments adorned with triangular spines; cuticular surface of cephalon and posterior portion of trunk segments richly adorned with small blunt spines/tubercles; cephalic gnathobases with elongate proximal “arm”; gnathobasic teeth differentiated antero-posteriorly (slender and long to short and stout); cephalic endopods with setal brush on podomeres 5 and 6; five-segmented thorax bearing strong biramous appendages with robust, clawed endopods and long basipods; very long (subequal to slightly greater than head and trunk length) bipartite telson, with a long, dentate proximal portion adorned with lateral spines, and a short distal portion about 1/3rd as long as proximal portion.

**Description.** *Habitus*. Body 8.5 to 34 mm (without telson), elongate, 19-segmented and tagmatized (Fig. 1): cephalon (seven segments with tergites fused in a single shield) (Figs. 1, 2a–g), trunk (12 segments) (Fig. 1), tailpiece (bipartite) (Fig. 1); trunk subdivided into tagma II/“mesosoma” (five anteriormost segments) and tagma III/“metasoma” (Fig. 1); “mesosoma” bearing robust walking legs (Fig. 1); tailpiece a very long (ca. length of cephalon and trunk) and slender spiniform telson with articulated terminal piece (Fig. 1f, g).

*Cephalon (“prosoma”)*. About 30% of trunk length; length about 80% of width (Fig. 1). Composed of a central, bulging area, housing the stomach, and of lateral pleural expansions of the tergal shield, sub-aligned with the frontal plane (Fig. 2g and Additional file 3, Additional file 4, Additional file 5, Additional file 6, Additional file 7). Broad and sub-triangular in dorsal view, with antero-medial margin separated from lateral (pleural) margins by strong ocular notches (Figs. 1b, 2a and Additional file 3, Additional file 4, Additional file 5). In lateral view, dorsal section of the cephalic shield high and sub-convex with relatively longer anterior face; ocular notch strongly impressed at the anterior junction between dorsal and postero-lateral sections of head shield; cephalic pleurae usually not visible in lateral view, due to their sub-horizontal position (Figs. 1, 2a, b). Pleural margins of the cephalic shield adorned with short, triangular spines (Fig. 2g and Additional file 6, Additional file 7); cephalic shield adorned on its entire surface with numerous, scattered short and blunt spines (Fig. 1b, h).

*Eyes*. Presence of a pair of spherical lateral eyes, presumably compound, inserted at the anterior notches between dorso-medial head bulge and lateral pleurae; approximately the size of the notches, i.e. 14% of head length in diameter; no trace of peduncle (Figs. 1a–d, 2a, g).

*Hypostome-labrum complex*. The anteriormost margin of the body bears medially a structural complex composed of dorsal and ventral elements, homologized here with labrum and hypostome.

*Labrum*. Anteriormost is a bulging body protrusion with length ca. 13% of head length, with rounded anterior margin slightly extending beyond head shield in dorsal view, and interpreted as a labrum (Fig. 2g, i).

*Hypostome*. In ventral view, a sclerite with possible midline separation beneath the frontal protrusion (labrum) is interpreted as a hypostome (Fig. 2g).

*Mouth*. Mouth opening located very anteriorly, above first pair of gnathobases, and opening ventrally (Figs. 1c, 2d).

*Alimentary tract*. Gut differentiated into foregut, stomach and intestine; foregut directed dorsally (“oe” in Fig. 2d), gently curving posteriorly towards a large stomach; stomach much wider than deep, occupying most of head space and partly housed within a dorsal bulge of the cephalon (Fig. 1d); stomach constricted at the posterior margin of cephalon to form the intestine, ca. 1/4 of body width, tapering to trunk segment 5, and finally reduced to a much smaller (ca. 1/3 of anterior diameter) duct in trunk segment 6 (Fig. 1c–g); anus opening posteriorly in anal pouch (Fig. 1a-d, g; see “tailpiece” section below).

*Cephalic appendages*. Post-ocular appendicular head composed of a pair of reduced frontal-most appendages, a series of branched antennule-like appendages and a series of five large, elongate, toothed, forward-oriented gnathobases bearing seven-segmented elongate spinose legs (Figs. 1, 2); an additional pair of appendages, with broad, rounded exopods and well-developed endopods, is located between the cephalon and trunk, but likely belongs to the cephalic tagma in the absence of dedicated trunk tergite (Figs. 1b, 2a, g and Additional file 5, Additional file 6).

*Frontal-most appendage pair.* Flexible (articulated?) and very short (about half the length of the following first gnathobasic appendage’s endopod) (Fig. 2i–m); termination unclear, claw possibly present (Fig. 2i).

*Post-frontal series of appendages.* Five pairs of hypertrophied gnathobases occupy most of ventral space under head shield (Figs. 1, 2); gnathobases with toothed, straight masticatory margin parallel to ventral margin concentrated ca. within the anterior first 40% of head length (in short succession), so that the portion between masticatory margin and attachment (“arm”) is increasingly elongated in posterior gnathobases—reaching up to ca. 50% of head shield length for appendage 5 (Figs. 1c, g, 2b, d); overall size of gnathobase also increasing posteriad, with anteriormost gnathobase inclusive of teeth roughly as wide as half the labrum (2B, D, G, L, N) and posteriormost gnathobase with length of masticatory margin around a 1/4th of head length (Fig. 1c-e, g); gnathobases extensively overlapping (Figs. 1c, g, 2b, l); masticatory margin with ca. 18 longer and shorter teeth arranged in two staggered rows, so that long and short teeth imbricate when opposing margins are closed; all teeth strongly sclerotized (Figs. 1c-e, g, 2d-f, n); both sets of teeth gradually decreasing in size proximalward; masticatory margin with convex latero-distal and latero-proximal margins; distalmost portion of masticatory margin pointing outward (Fig. 1e); from gnathobasic appendage 3 to 1, teeth increasingly more slender, sharper and longer in relative size, so that the anteriormost gnathobase displays a morphology (short armature with long and slender teeth) substantially different from posteriormost (long armature with shorter, blunt, more robust teeth) (Fig. 2d, n).

Endopods seven-segmented, gradually increasing in size posteriorward, from 40% of head length to twice this size, with a distinctly larger increase between legs 3 and 4 (Figs. 1c, d, 2g–m); bent proximally and forming an almost 90 degree angle between podomeres 2 and 4; podomere size formula is [x / x+(1/5)x / x+(2/5)x / x+(1/5)x / x+(2/5)x / x / x (claw)] proximo-distally; terminal podomere a strong claw with two successive teeth on its ventral margin and adjoined by a pair of smaller, slender claws posteriorly (Fig. 2k); at least podomeres 2 and 3 bear well-developed enditic projections on their distalmost margins, forming platforms where insert bundles of more than 9 setae, of which the three central ones are more robust (Fig. 2j); proximalmost podomere (1) of each endopod inserts on distal margin of corresponding gnathobase, close to the distal border of the masticatory margin (Fig. 2n).

Exopods are represented by five long and slender rami made of seven or more podomeres (Figs. 1, 2); each segmental junction bears three stiff setae (Fig. 2c); distalmost unit bears at least three setae; podomeres with diameter distinctly reduced proximal-ward and sub-concave margins (Fig. 2c); length of each subsequent podomere is ca. 20% greater than preceding podomere (Fig. 1a); all rami increasing gradually in size posteriorward, but first ramus distinctly shorter, only slightly longer than endopod of first gnathobasic appendage (Fig. 2i); attachment unclear, but does not appear to be on main branch of gnathobasic appendages; fifth ramus apart from the others in dorsal view and projecting more laterally close to the endopod of the fifth gnathobasic appendage, suggesting a different attachment (Fig. 2g and Additional file 3, Additional file 5, Additional file 7).

*7th head appendage*. One pair of biramous appendages located at junction between cephalon and trunk (“ia” in Figs. 1a, b, 2a, g, 3a); morphology similar to thoracic appendages, but reduced to ca. 2/3 of length of first three trunk pairs; exopod more circular with finer and more numerous lamellae (Figs. 1b, 2a and Additional files).

*Trunk (“opisthosoma”)*. *Tergo-pleurae*. 12-segmented, anatomically divided into anterior (five first segments, thorax or “mesosoma”) and posterior (post-thorax or “metasoma”) tagmata (Fig. 1); segments forming strongly bipartite tergo-pleurae, with an anterior portion (“doublure”) mostly unadorned on its surface and a stronger, elevated posterior portion (“armature”) covered in short blunt spines (similar to the surface of head shield) and produced into paired dorsal projections adorned with longer and sharper spines (Fig. 1); upper antero-lateral margin of armature fused with doublure, so that the discontinuity between doublure and armature is more pronounced on pleura and posterior side of segment (Additional files 3, 4); margin of doublure produced on its pleural circumference into sharp lanceolate spines growing more elongate distalward, with longest spines (ca. 2/3rd of segment length) on the posterior side of pleural distal extremity (Fig. 1b–g); margin of pleural part of armature adorned with minute spines; length of upper doublure less than half armature length in anterior segments but increasing posteriad to reach about identical length with armature; doublure width reducing on anterior side of pleura up to fusing with armature margin; on posterior side of pleura, margin of doublure closely parallel to margin of armature (Fig. 1b–g); anterior pleurae sub-horizontal, from segment 3 posteriorward gradually more parallel to body axis; pleura of first segment about 3/4th of cephalic pleura length, pleura of second segment subequal to cephalic pleura, pleura of third segment slightly longer than cephalic pleura, posterior pleurae gradually decreasing in size, except for pleura of last segment, forming an elongate blunt blade reaching back of telson base (Fig. 1b–g); margins of doublure and armature also increasingly curved posteriad.

*Thoracic appendages*. Biramous with well-developed endopods, present in trunk segments 1 to 5 (Fig. 1); appendages in segments 1–3 subequal, attachment to tip of endopod about 120% of head length; endopods of appendages 4 and 5 distinctly longer than 1–3, with endopod 5 also slightly longer than 4 (Fig. 1 and Additional files 5, Additional file 6, Additional file 7).

Basipod broad and cylindrical, ca. 1/3rd of endopod length (Fig. 1b and Additional file 6).

Endopod seven-segmented (six podomeres plus a claw); first (proximalmost) podomere quadrate, podomere size formula [x / x / x+(1/3)x / x+(1/4)x / x+(1/3)x / x+(1/2)x / (3/4)x (claw)] proximo-distally (Fig. 1a-c, g and Additional files 4, Additional file 5, Additional file 6, Additional file 7); mesio-distal margins of podomeres forming thickened rims; terminal claw strong, length about half of podomere 6, adjoined posteriorly by two smaller slender claws (Fig. 1a and Additional files 4, Additional file 5, Additional file 6, Additional file 7).

Exopods paddle-like, margins fringed with thin lamellar setae except on the most proximal left and right thirds; paddle connected to an attachment podomere inserted via an elongate hinge on the distal half of the basipod; exopod length about 2/3rd of endopod (Figs. 1b, 2a and Additional file 6).

*Post-thoracic appendages*. Broad, sub-spherical exopods slightly jutting out beneath pleurae from segment 6 to 12; exopod size decreasing gradually to segment 11 (Fig. 2h); exopod of last segment much smaller; no adornment visible; endopods presumably reduced or absent.

*Tailpiece*. Tailpiece a very long (subequal to slightly longer than head and trunk combined), bipartite, telson with spinose lateral margins (Fig. 1); first piece composed of a broad “base” (telson head) on the first 1/9th of its length and of an elongate, much thinner posterior rod-like extension with gentle dorsal concave curvature (Fig. 1a–g); on dorsal side of base, parallel carinae converging from positions of trunk protrusions on trunk tergites and running entire length of first telson piece (Fig. 1a–g); ventral side of base protruding, with sharp anterior slope, sub-straight margin pointing dorso-posteriorly and curved margin joining with more slender part of telson piece (Fig. 1a, b); an oblong cuticular structure, the anal pouch, is attached to sub-straight margin of some specimens, pointing postero-ventrally (Fig. 1); length of anal pouch about 12% of length of first telson piece; lateral spines sharp, decreasing in size posteriad, all successive except for first two spines on base closer to one another than to following spine, itself followed by a gap before the next; second telson piece a straight articulating rod about 1/3rd of first telson piece length and ending in a set of three setae (Fig. 1f).

**Remarks.** We found *Habelia brevicauda* [38] to belong outside habeliidans, and the revision of this taxon is the object of a current study.

***Habelia optata*** Walcott, 1912.

**Synonymy**

- 1912 Walcott, pp. 202–203, pl. 29, fig. 6
- 1920 Raymond, pp. 120–121
- 1920 Henricksen, pp. 16
- 1944 Størmer, p. 86
- 1959 Størmer, *in* Moore, p. 31, figs. 19, 3 (copy of Walcott (1912))
- 1964 Simonetta, pp. 219–222, fig. 2, pl. xxxvi, unnumbered figures of USNM 57693, 139,209, 144,907–909
- 1975 Simonetta and Delle Cave, pp. 27, 32, pl. iii, fig 2a, b (reproduced from Simonetta (1964)), pl. xxi, figs. 1-3, figures of same specimens as Simonetta (1964)
- 1981 Whittington, pp. 343–346, fig. 61; figs. 62–66, plate 7; figs. 67–71, plate 8; figs. 72, 75–77; figs. 78–83, plate 9, fig. 130

**Diagnosis.** As per genus. Morph A (Figs. 1d-g, 3, 4 and Additional files 4A–D, 7E, F, J, K): First three anterior dorsal trunk projections long, with first longest (about 45% of head length), second slightly shorter than first and third slightly shorter than second; first projection pointing dorsally and other two pointing increasingly posteriad; antero-proximal portion of projections sub-straight, so that distal portions of projections 2 and 3 form an obtuse angle with that base; first pair of projections with antero-proximal base forming convex margin in lateral view; posterior dorsal projections convex dorsally, rounded anteriorly and pointed posteriorly. Morph B (Fig. 1a-c, h and Additional files 3, Additional file 5, Additional file 6, 7A–D, G-I): Five anterior-most pairs of dorsal spinose elevations forming rounded mounts decreasing in size posteriad and differentiated so that the pointed posterior end is moderately raised dorsalward: angle of posterior spinose projection with longitudinal plane of body ca. 70° in ts6, 80° in ts5 (but with more rounded aspect) and 85° in ts4–1; ornamental spines stronger, longer and more numerous on ts1–5; posterior dorsal projections convex dorsally, rounded anteriorly and pointed posteriorly.

**Description.** As per genus and diagnosis.

**Remarks.** Morphs A and B are clearly discriminated by the presence of anterior dorsal spines, with no indication of intermediaries, and no obvious relationship to overall size. This dichotomy does not overlap with the presence of “anal pouch,” as the latter can be either present or absent in specimens with dorsal spines. It is worth noting that specimens with and without elongate trunk spines as well as specimens with or without anal pouch co-occur on the same stratigraphic levels within the Walcott quarry (Additional file 1). This supports the idea, on the basis of niche distribution, that these traits are conspecific, and that one of them likely characterizes sexual dimorphism. For this reason, we refrain from erecting distinct morphospecies based on these morphs, and both are considered for now to belong to *H. optata*.

### Phylogenetic results

**Fig. 5:**
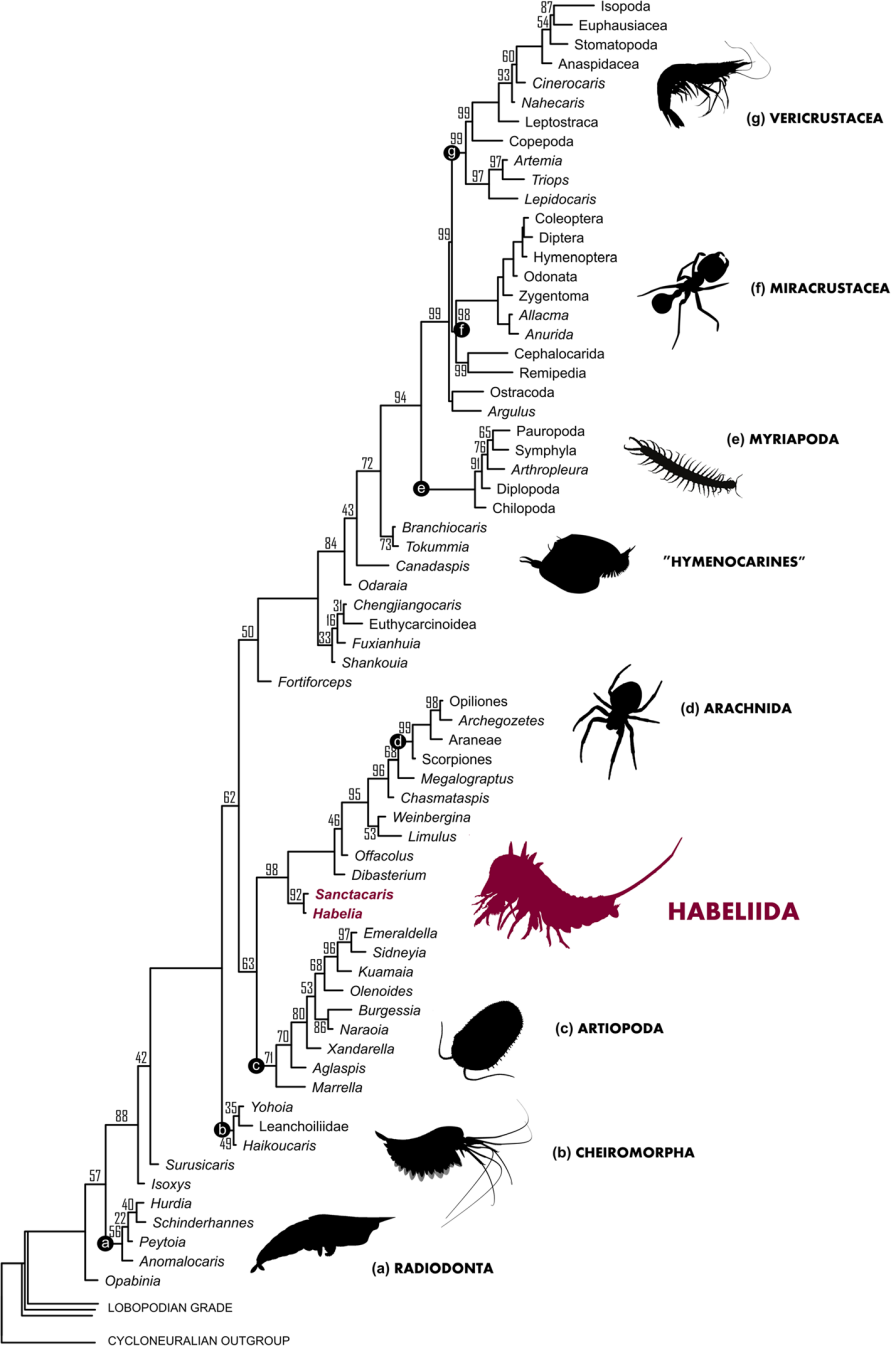
Maximum clade credibility tree of a Bayesian analysis of arthropod relationships, using an Mkv model on a morphological matrix of 77 taxa and 215 characters. Habeliidans are in bold and ***red***. Numbers next to nodes are posterior probabilities when <100

Our Bayesian analysis finds *Habelia* and *Sanctacaris* grouped in a clade at the base of Chelicerata (Fig. 5). The Chelicerata clade is composed of paraphyletic “merostomes” (xiphosurans, eurypterids and chasmataspidids), from which emerges a monophyletic Arachnida. This topological arrangement is broadly consistent with the latest fossil-inclusive studies investigating chelicerate relationships [30, 56]. *Contra* [30], however, we find *Offacolus* and *Dibasterium* to lie as sister taxa to all other euchelicerates (as in [56, 57]), in contiguity with habeliidans.

In the topology presented here, Chelicerata is equivalent to Euchelicerata because we removed Pycnogonida from the analysis. The phylogenetic position of pycnogonids has long been an issue [47, 51, 58], and morphologically the presence of uniramous endopods (derived for euchelicerates) with a (possibly) four-segmented head tagma (plesiomorphic for arachnomorphs) are character states that are highly conflicting in this particular topology—especially with the inclusion of *Habelia*. Because of this, coding pycnogonids with a four-segmented head places them with *Marrella* at the base of the Artiopoda, whereas a seven-segmented coding affects the position of *Offacolus* and *Dibasterium*, bringing *Limulus* and *Weinbergina* as basalmost (due to their loss of exopods). We consider hereafter that pycnogonids still define Chelicerata as sister group to euchelicerates (i.e., branching immediately following habeliidans), but their exact phylogenetic placement will require further investigation.

In concordance with [56, 57], we retrieve *Chasmataspis* and *Megalograptus* as paraphyletic, but *Limulus* and *Weinbergina* as forming a clade. This could be due to our limited taxon sampling. The retrieval of cheiromorphs as part of a merostome clade by Garwood and Dunlop [30] may illustrate a problem of character polarization across higher nodes.

A notable difference with the previous analysis of this dataset under parsimony [9] is the retrieval of Arachnomorpha sensu Störmer [36], that is, the monophyletic group composed of Trilobitomorpha sensu lato (or Artiopoda sensu Hou and Bergström [4]) and Chelicerata. It has been recently shown that Arachnomorpha could result from a polarization bias overweighting the absence of characters in a dataset, for instance when coding inapplicable entries as additional states [27]. The fact that we retrieve here this clade using a Bayesian approach instead of parsimony, and after coding inapplicable entries as uncertainties, suggests that the grouping of trilobitomorphs with chelicerates excluding cheiromorphs (*contra* Arachnomorpha retrieved in refs. [6, 27]) is a resilient configuration. Such resilience to methodological variations is further supported by the fact that these taxa have also been found to be monophyletic using implied weighting [8].

Additionally, the probabilistic approach does not support the monophyly of Hymenocarina and Megacheira. The critical uncertainties (e.g. presence of mandible) in the coding of large bivalved taxa related to protocaridids are likely responsible for the dissolution of Hymenocarina compared to the parsimony-based topology, since character state transformations are optimized differently under a Bayesian treatment. The redescription of these taxa in light of the new anatomical information provided by *Tokummia* and *Branchiocaris* [9], as well the detailed redescription of *Waptia* currently in progress, should help clarify the validity of Hymenocarina as a clade. The status of Megacheira is likewise dependent on the reevaluation of “multisegmented” forms such as *Fortiforceps* [4] with respect to the better-diagnosed Cheiromorpha [27].

The principal characters involved in the resolution of habeliidans are presented in Table 1. The full development of endopods in the head tagma, presence of strong gnathobasic basipods and tripartite apoteles constitute the core characters supporting Arachnomorpha in our matrix. This result guided our revision of the arachnomorph diagnosis above. The positioning of habeliidans within “panchelicerates” is necessarily based on new or redefined characters constituting original apomorphies for this expanded chelicerate clade. We found that a seven-segmented cephalon (or prosoma) and the modification of posterior trunk appendages initially involving the complete reduction of the endopod are characters potentially diagnostic of the “panchelicerate” or total-group Chelicerata clade and plesiomorphic conditions of Chelicerata. The absence of chelicerae separates habeliidans from chelicerates, but the chelate condition is still uncertain in habeliidans and thus this exclusion should be regarded as provisional. It is also possible that the frontalmost endopods of habeliidans have a distal morphology intermediate between antennule and chelicera, which will require a revision of the definition of the chelicera itself. Euchelicerates remain securely established by the presence, as part of their ground pattern, of opisthosomal opercula.

**Table 1:** Main diagnostic characters for clades inclusive of or related to habeliidans, and remarks on their significance. Potentially important characters with ambiguous optimization on the tree are italicized

Clade	Character	Remark
Arachnomorpha	All cephalic endopods fully developed (char. 81)	“Panchelicerates” and artiopodans are characterized by having well-developed endopods—based on a heptopodomeran ground pattern [27]—in their head tagmata. In mandibulates, at least one of these endopods is usually strongly modified; in leanchoiliids, the first post-frontal endopod is likely reduced [27, 67], but the condition is not well known in other megacheirans.
	Third cephalic appendage gnathobasic (char. 107)	We use here the term gnathobasic for a basipod with well-developed gnathal (usually dentate) edge on its proximal margin, without presence of a coxa. This is a possible ground pattern of Arachnomorpha.
	Presence of gnathobase(s) (char. 177)	By extension, the presence of a masticatory gnathobase on any body limb is another possible synapomorphy of arachnomorphs. This would not support the placement of *Marrella* [103] at the base of Artiopoda, but the proximal limb morphology in marellomorphs needs to be investigated in more detail.
	Trunk endopods ending in set of three claws (“apotele”) (char. 202)	Although the arrangement of the three terminal claws may vary, the tripartite apotele has already been presented as a potential synapomorphy of Arachnomorpha [104]. The claw complex seen in the thoracic endopods of habeliidans is consistent with this view.
	*Posteriormost trunk tergites fused into single plate* (char. 212)	Given our topology (Fig. 5), the thoracetron of xiphosurids and the pygidia of trilobites or other trilobitomorphs are not directly inherited from a common ancestor. The fact, however, that these structures are only found in arachnomorph arthropods suggests that the corresponding genetic pathways are shared and a possible case of parallelism.
“Panchelicerata”	Ground pattern of a seven-segmented prosoma (chars. 32)	We construe that in habeliidans, as in other xiphosurans [62, 66], the seventh appendage pair in the head is homologous to the chilaria. We also co-opt here the hypothesis that the “antennular” appendages of habeliidans are modified exopods of the head limbs, as previously interpreted in *Sanctacaris* [26, 32].
	Trunk appendages with reduced or vestigial endopods (char. 183)	In this study, we propose that the absence of endopods on the posterior trunk appendages of habeliidans is an ancestral condition related to the reduction of biramous trunk appendages in chelicerates. In many cases, trunk appendages are still present among euchelicerates in vestigial form, such as spinnerets, ventral sacs, gonopods or genital acertabula [105].
	*Labrum* (char. 58)	The presence and homology of a “labrum” remains ambiguous in higher nodes of euarthropods, but remains diagnostic of “panchelicerate” (as shown herein) and mandibulate taxa. We propose here that the soft dorsal structure observed in habeliidans is equivalent to the soft elements identified underneath the frontal sclerite of protocaridids [9].
	*Differentiation of the seventh prosomal appendage* (char. 149)	The value of this character depends on the semantic boundary assigned to “differentiated.” We did not consider here that the seventh pair of appendages in habeliidans or *Weinbergina* was already differentiated compared to other trunk limbs. Ideally, this character will be refined using a more precise statement of differentiation, for the diagnosis of either Panchelicerata or Euchelicerata.
Chelicerata	Chelicerae (char. 73)	The chelate condition of the reduced frontalmost endopods of habeliidans is uncertain. However, contrarily to other characters evaluated here, the presence of chelicerae is the defining condition of Chelicerata, and therefore this clade could be enlarged in the future.
	*Fused post-oral ganglia* (char. 47)	Whether this character can be coded in pycnogonids is not clear [106]. A single post-oral nerve mass has been interpreted in a leanchoiliid from China [29], but it appears to us that the central nervous system cannot be clearly isolated from other tissues in their specimen (such as cephalic shield and appendages), and thus the origin of this condition remains uncertain.
Euchelicerata	Opercula on ventral surface of trunk (opisthosoma) (char. 151)	The presence of ventral opisthosomal plates called opercula has been shown to be a likely apomorphy of euchelicerates [65], which is supported herein. No evidence of elements possibly homologous to opercula have been found in habeliidans, although we did not have access to a clear ventral view of the trunk.
	*Post-frontal appendage with chelate or subchelate termination (char. 93)*	Given the basal phylogenetic position of *Offacolus*, *Dibasterium* and xiphosurids, a chelate or subchelate pedipalp (or walking leg in xiphosurans) may be considered a groundplan character of Euchelicerata. However, this condition is clearly highly convergent in euchelicerates overall, and whether it represents broad parallelism bears on the resolution and morphology of synziphosurines at the base of euchelicerates.
	*Endosternum* (char. 55)	The euchelicerate endosternum is of course difficult to document in fossils, which hampers an assessment of its origin.

## Discussion

### Significance of morphology

Several authors [5, 32, 38] have recognized in habeliidans the peculiar bundled aspect of preservation of the anterior limbs. In *Habelia*, this configuration is associated with the presence, posterior to the frontal bundle, of unusually large gnathobases (Figs. 1c-e, g, 2b–n and Additional files 4, 7). These five gnathobases occupy most of the space under the cephalic shield, leaving no room for the insertion of other appendages. This condition, consistent with the anterior and horizontal position of the masticatory margins of those bases (Figs. 1c, g, 2d and Additional file 7), and the number and position of the “bundled” spinose limbs (Figs. 1a–d, 2b–n and Additional files 4, Additional file 5, Additional file 6, Additional file 7), must lead to the conclusion that the latter are endopods inserted distally, close to those toothed margins. In some cases, the attachment of the endopods on the gnathobasic basipods has been preserved (Fig. 2b, n). There is also evidence of a pair of appendages posterior to the gnathobases that seems to be inserted at the junction between the cephalon and trunk (Figs. 1a, b, 2a, g and Additional files 5, 6), and which would bear a large exopod adorned with very thin lamellae (Additional file 6). Such appendage is not dissimilar to what is known in the head of *Sanctacaris* (Additional files 1 and Additional file 9). The peculiar position of cephalic endopods in habeliidans is therefore genuine, and is due to the distal attachment of these endopods onto gnathobases increasingly large posteriad, occupying the seemingly unoccupied space under the cephalic shield.

In addition, there is a pair of reduced appendages anterior to the gnathobase-bearing limbs (Fig. 2i, M and Additional file 5). These frontalmost, reduced endopods occupy a topological position corresponding to the chelicerae, and are thus regarded as their likely precursors. At present, it is uncertain whether these appendages are chelate in *Habelia*, and they are not known in *Sanctacaris* (see Additional file 1). Our phylogeny (Fig. 5) does not allow conjecturing through the reconstruction of ancestral states either, as the placement of pycnogonids is uncertain (see Methods) and they are not included in our final analysis. From a functional point of view, the presence of reduced clawed appendages at the front may seem at odds with the association of the raptorial “bundle” of exopods and the gnathobases, much more efficient in grabbing and dissecting food items. Thus, for now, we refrain from formally assigning habeliidans to Chelicerata, eponymously defined by the presence of chelicerae (Table 1).

The “protochelicerae” at the front of *Habelia*’s head flank a medial dome-shaped structure (Fig. 2g, i and Additional file 5). In dorsal view, this element shows no sign of suture, partial detachment or doublure, and its preservation lacks three-dimensionality. We do not think, therefore, that this is a sclerite, but rather a protrusion of the body. This soft protrusion is positioned just in front of the mouth, and thus conforms best to the labrum of chelicerates [59, 60]. In ventral view, a more strongly sclerotized structure is preserved three-dimensionally underneath the labrum, and likely has a similar shape. We tentatively homologize this ventral pre-oral sclerite with the hypostome of artiopodans and other pre-oral plates in extant groups. Such “hypostomo-labral complex” in habeliidans also calls for a comparison with a similar set of frontalmost features described in a number of hymenocarine mandibulates [9]. Since the frontal sclerite in protocaridids and *Canadaspis* is dorsal, instead of ventral, it probably does not correspond to the hypostome-like plate seen in *Habelia*. The feature shared by those taxa would therefore be the soft protrusion, or labrum per se, which may be bipartite in protocaridids [9]. The homology of the inter-ocular lobes in *Canadaspis* remains unclear, but it is worth noting that they occupy the same para-labral position as the “protochelicerae” of *Habelia*.

In *Habelia*, the frontalmost appendages, the five pairs of gnathobasic appendages and the thoracic-like biramous appendages inserted at the back of the head therefore lead to the formation of a cephalic tagma encompassing seven pairs of appendages. Although most chelicerates are diagnosed by the possession of a six-segmented prosoma [28], the chilaria of xiphosurids and the possible implementation of a seventh pair in *Weinbergina* had raised the question of a seven-segmented prosoma possibly representing the chelicerate ground pattern [61]. This hypothesis was strengthened by the publication of *Offacolus*, a possible stem euchelicerate also sporting a seventh (and differentiated) appendage under its head shield [62]. The opisthosomal origin of the chilaria, demonstrated both embryologically [63] and morphologically [64], however, suggested that the integration of that first opisthosomal limb into the prosoma could be a derived character present in all “xiphosuran” taxa.

The anatomy of habeliidans shows that this condition is present outside of the “xiphosuran” body plan and our topology (Fig. 5) suggests that a cephalon with seven segments (i.e. eight somites) resolves as a plesiomorphic condition of Chelicerata. This means that the studies documenting the opisthosomal affinity of the seventh prosomal appendage [63, 64] were in fact providing atavistic evidence for a transformation that occurred in the ancestor of all “panchelicerates,” and possibly reflecting a morphological variability present at the origin of arachnomorphs. In this evolutionary context, *Habelia* allows us to link the xiphosurid chilaria [65] to a fully-formed, opisthosoma-like biramous limb. In arachnids, this somite is usually considered the first of the opisthosoma, but shows a variety of morphological differentiations, such as a constriction associated with a reduced tergite—as is the case in Araneae [28]. As we discuss below, the biramous limb corresponding to this somite in habeliidans stems itself from a typical cheiromorph limb.

The “antenna-like rami” of *Sanctacaris*, as Briggs and Collins [32] originally called them, have been recently compared to the stenopodous exopods of the “xiphosurans” *Offacolus* and *Dibasterium* [26]. These frontal appendages in *Sanctacaris* and *Habelia* are in fact quite distinct morphologically from the exopods of *Offacolus* and *Dibasterium*, with long and slender podomeres, giving them indeed a more “antennular” aspect. Similar to at least *Dibasterium* (but also likely *Offacolus* [66]), however, these rami in *Habelia* are preserved separately from the endopod “bundle” in non dorso-ventrally-preserved specimens, suggesting that they do not attach to the limb basis like regular exopods. In *Dibasterium*, the basipod itself was reported absent [66]. Notwithstanding the phylogenetic placement of pycnogonids and its impact on the polarization of characters on the tree (see (b) Phylogenetic results), this condition may constitute a strong argument that *Offacolus* and *Dibasterium* are indeed basal taxa [56, 57], close to habeliidans.

Specimens of *Habelia* preserve the first four of these long anterior rami also bundled together, and sometimes even apparently attached at their base, except for the fifth one preserved more posteriorly and pointing laterally (Figs. 1c, 2g, and Additional files 3, 5, 7). If they indeed represent dissociated exopods, their possible partial fusion at the base would be secondarily acquired, rather than demonstrating any relationship with “great appendages” or any other appendage pair belonging to its own somite. The anatomy and morphology of habeliidans thus challenge the direct morphological continuity between cheiromorph “great appendages” and chelicerae as proposed by some authors [24, 25] in favour of an intermediate, reduced state that may not have been chelate.

There are, however, shared characters between megacheirans and habeliidans, which not only have an important impact on phylogenetic relationships but also, consequently, on the alignment of the various euarthropod body plans. One of our main findings is that *Habelia* bears thoracic appendages of typical cheiromorph morphology [24, 25, 27, 67, 68]: long, subcylindrical, non-gnathobasic basipods, to which are attached a two-segmented, paddle-like exopod fringed with oblanceolate lamellae and a seven-segmented endopod with limited proximo-distal podomere differentiation (Fig. 1a-c, g, 2a and Additional files 4, Additional file 5, Additional file 6, Additional file 7). This condition in *Habelia* (and, potentially, in habeliidans) conflicts with the existence of a stem lineage to chelicerates composed of artiopodans (as in ref. [26]), because such a hypothesis would imply a major reversal of gnathobasic limbs with differentiated exopods through all trunk segments. Hence the opisthosomal limbs of chelicerates might have never been gnathobasic, and some in-group apomorphies might well be plesiomorphies instead. This is the case, for example, for the “13 opisthosomal segments” character used by Lamsdell [69] to define the clade “Dekatriata.” Counting the “release” of the seventh pair of cephalic appendages, this character was already defining habeliidans, while 12 trunk segments were present in a cheiromorph such as *Yawunik* [27].

**Fig. 6:**
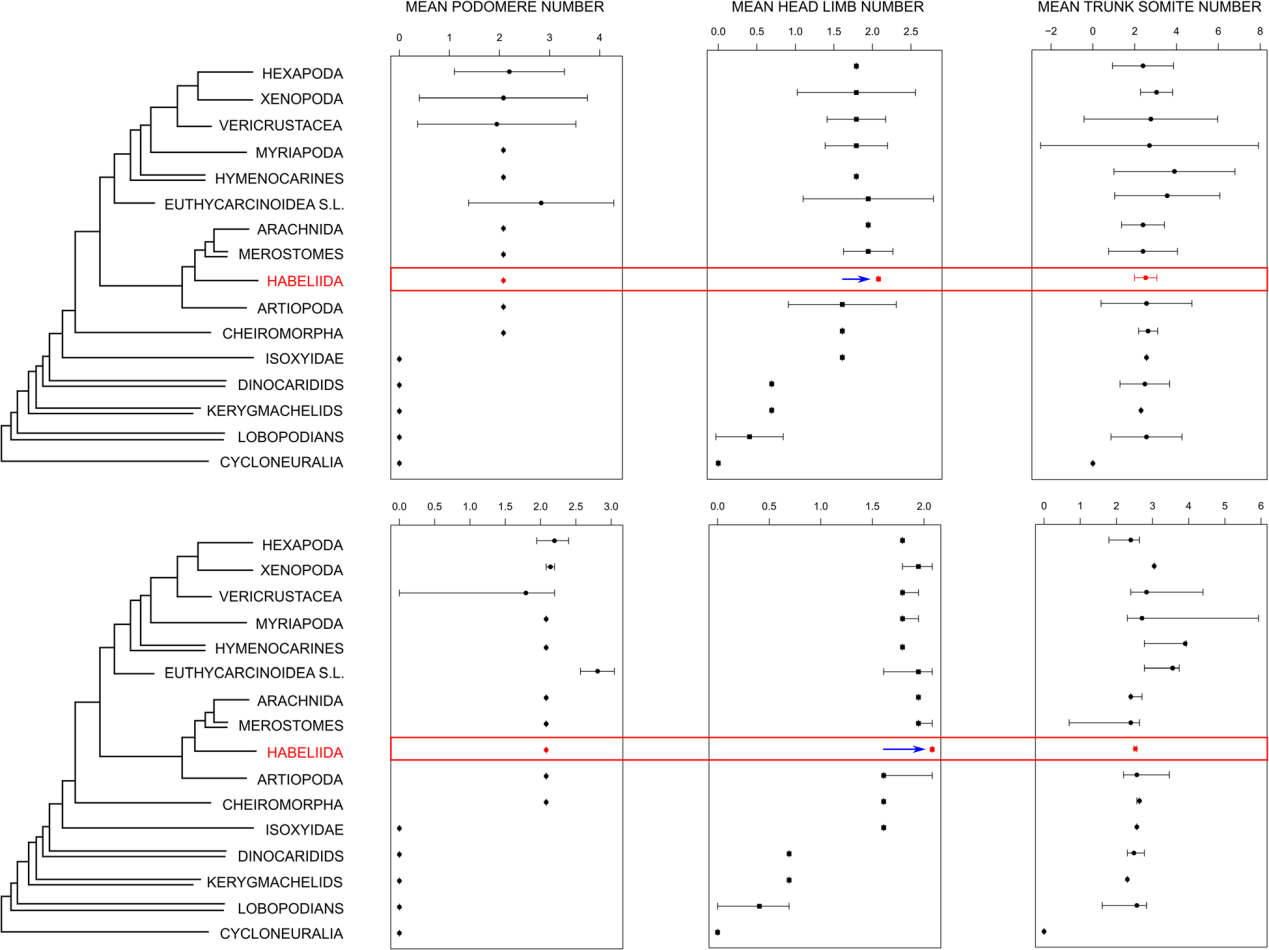
Segmental composition of major panarthropod groups as expressed through the mean of numbers of podomere (left), head limbs (center) and trunk somites (right). We use logarithmic instead of raw values to facilitate reading. Upper graphs have their error bars representing the standard deviation of the data; the error bars for lower graphs represent the minimal and maximal values inside each group. Note the punctual effect of the evolution of habeliidan heads (blue arrows) compared to the trend observed for the trunk. Tentatively, we have considered the first maxilliped in certain mandibulates as part of the functional “head,” owing to its high morphological integration to that tagma. Trilobites are not included here, their high plasticity in trunk somite number being considered autapomorphic and an arguable deviation from the general pattern seen in other artiopodans. The topology is based on phylogenetic results presented herein (Fig. 5). Double branches indicate paraphyletic groups

The direct implication for the alignment of the “panchelicerate” and cheiromorph trunks is that a large discrepancy between head tagma arises. Given that the plesiomorphic head tagma consists of four segments (as in isoxyids [44] and megacheirans [27]), the two or three added pairs must have been incorporated to the cephalon from existing trunk segments. However, as mentioned, the number of trunk segments in this part of the arthropod tree is rather well conserved in adults, with documented variations of only one or two segments (Fig. 6). This implies the insertion of up to three additional somites within the head either de novo or from the trunk with a subsequent adjustment of the total number of trunk somites. In both cases, such an anatomical transition could represent an event of punctuated equilibrium [70], as already reported for instance in certain centipedes with single speciation events involving the addition of numerous somites [71].

Xenopodans—represented by *Sidneyia* and *Emeraldella*—have long been suggested as possible links to chelicerates from trilobitomorph ancestors [26, 36]. However, the head composition of *Sidneyia* remains mostly unknown [72], while *Emeraldella*, described as having a ground-pattern type of head tagma with four appendage pairs [73], is more likely to have five cephalic pairs, including antennules, and a trunk of 12 segments. Perhaps more compelling is *Xanderalla spectaculum* Hou et al. [74] from the Chengjiang biota, with a 12-segmented trunk and a set of six post-antennular cephalic limbs, the last of which is close to the articulation with the first trunk segment [4]. Interestingly, the cephalon of *Xandarella* retains moulting sutures isolating an anteriormost section made of the antennules plus three appendage pairs [4], typical, by contrast, of the arthropod ground pattern [44, 75, 76].

By retrieving Artiopoda and “Panchelicerata” as two separate clades, our current cladogram (Figs. 5, 6) thus favours morphological variability and possible parallelism over gradual acquisition of a chelicerate-like prosoma. This topological configuration is notably supported by the plesiomorphic condition of habeliidan trunk appendages. In this context, the common arachnomorph ancestor probably had a head tagma composed of five or more somites, but had acquired developmental plasticity in the formation of the anterior tagma. Given the constraint in trunk somite number observed in closely related taxa (but released in trilobites [77]), such plasticity possibly involved the addition of de novo somites directly to the head. Pycnogonids, which also include variations in appendage number among taxa [28], could be representative of such selective release of developmental canalization in basal arachnomorphs.

Considering the morphological differences between the frontalmost appendages of megacheirans, artiopodans and habeliidans, and assuming homology between these, the frontalmost appendages of the arachnomorph ancestor is difficult to reconstruct. However, we can hypothesize an intermediate morphology in the form of a short, antennular, monobranch “great appendage”; a condition which could have given rise to both the long antennules of most artiopodans and the reduced “protocheliceral” appendages of habeliidans. Fossil euarthropods such as *Fortiforceps* [4], *Jianfengia* [78] and *Kiisortoqia* [79] bear frontalmost appendages that could be representative of such intermediate condition.

The rear tagma of *Habelia* (“metasoma”) bears pairs of well-developed rounded exopods, with no obvious sub-structure—only poorly-defined traces on posterior appendages that we can, at best, interpret as folds. Hence the specimens provide no evidence of the presence of lamellate gills characteristic of chelicerates, or of an equivalent of opisthosomal opercula [65]. There is also no trace of accompanying endopods, however, suggesting they may be reduced/vestigial, which so far has been also a diagnostic trait of Chelicerata. It appears therefore that the reduction of posterior endopods came first in the evolution of “panchelicerates,” leaving the metasoma possibly specialized for respiratory functions, although the surface of gaseous exchanges cannot be determined yet with *Habelia* (it may have been the surface of the exopods themselves). We assume that these exopods were later modified to become the gill-bearing opercula of euchelicerates.

A peculiar aspect of the morphology of *Habelia* is the rich adornment of the body—not seen in *Sanctacaris*. The only equivalents for such cuticular differentiations amongst Cambrian arthropods are the prosopon of trilobites, especially in their tubercular forms [80]. This could be seen as evidence reinforcing the monophyly of Arachnomorpha. Another characteristic that to some extent can be observed in trilobites is the strong differentiation of individual trunk segments between anterior and posterior parts. Such a phenotype is reminiscent of parasegmental patterns. Parasegmental development, which in extant taxa is known to be regulated by the pair-rule and patterning genes *fushi-tarazu*, *even-skipped*, *engrailed* and *wingless* at segmental boundaries [81], represent fundamental subdivisions of the post-cephalic development in euarthropods, prior to the consolidation of the somites as the final metameric units. In some cases, parasegmental boundaries express morphological differentiation in early embryos, such as in the opisthosoma of the model spider *Cupiennius salei* [81]. Given its posterior affinity and its strong impact on development, it is possibly the same mechanism that is at the origin of the tergo-pleural subdivisions in the trunk of *Habelia*. This would mean that part of the downstream regulation replacing parasegmental patterns would be inactivated in this taxon. If so, it would be reasonable to think that other aspects of the development might have been altered (or “relaxed”), especially with respect to the head tagma.

### Palaeoecological implications

Although the masticatory margins of *Habelia*’s cephalic gnathobases bear similarities to those of other arachnomorphs, especially those of *Sidneyia*’s trunk limbs [72, 82], their relative size, posteriad increase of absolute size, shape and orientation (i.e., sub-parallel to the frontal plane of the head) make them unique amongst all known arthropods.

**Fig. 7:**
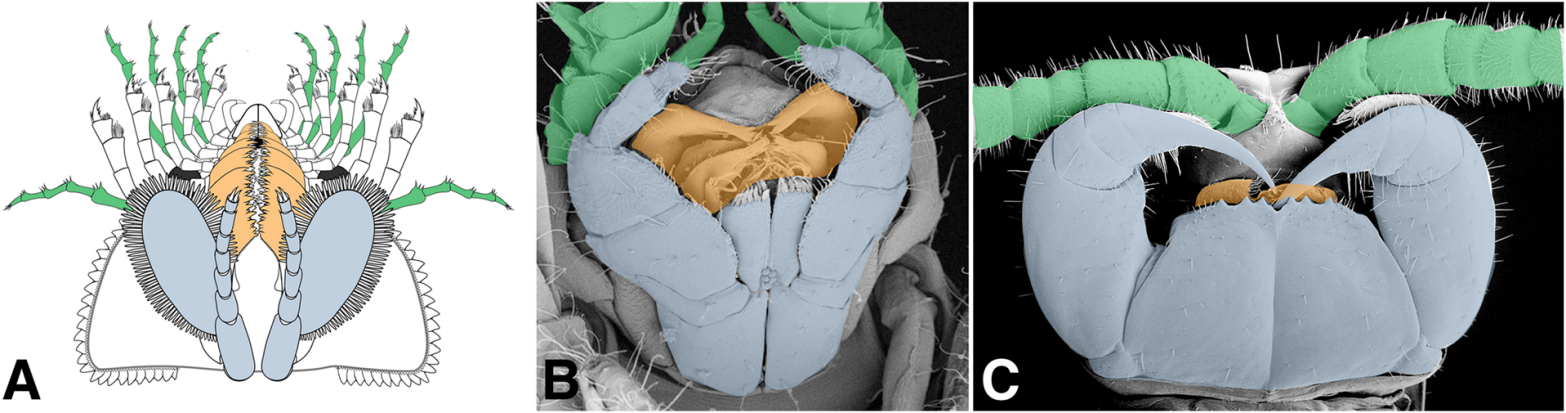
Convergences in head anatomy and morphology between *Habelia* (**a**) and selected mandibulates, in this case *Ianiropsis* sp. (Malacostraca: Isopoda; **b**; © Buz Wilson, Australian Museum) and *Henicops washpoolensis* (Myriapoda: Chilopoda; **c**; image provided by G. Edgecombe). Colours highlight the morpho-functional correspondence between sensory appendages (exopods in *Habelia* vs. antennae in mandibulates; ***green***), masticatory appendages (gnathobases in *Habelia* vs. mandibles and maxillae in mandibulates; ***orange***) and complimentary appendages aiding in food manipulation (seventh head appendage in *Habelia* vs. maxillipeds in mandibulates; ***blue***). Note that masticatory appendages in *Henicops* are hidden by the large coxosternites of the maxillipeds

In fact, the best functional analogs for dentate masticatory margins opposing parallel to the antero-posterior axis would be the mandible and other masticatory apparatuses present in the more differentiated heads of mandibulates (Fig. 7). In malacostracans and terrestrial mandibulates in particular, mandible, maxillule, maxilla and sometimes maxillipeds’ proximal podomeres also often form a succession of strong crushing sclerites often aided by modified endopods (palps) to constitute very efficient chewing devices capable of dissecting hard chitinous, sometimes mineralized cuticles or shells, in the vicinity of the mouth opening [83, 84]. This implies that at least some stem chelicerates could have occupied a typically benthic malacostracan niche in Cambrian marine ecosystems.

As a fundamental distinction, gnathal structures in mandibulates are derived from coxae, themselves being additional basal podomeres originating from the development of basipod endites [9, 85–89], while artiopodan and chelicerate gnathobases are transformations of the basipod itself. Furthermore, the successive appendages are morphologically near identical in habeliidans (except for variation in overall size as well as tooth length), while they are most of the time individually differentiated in mandibulates, as a means of performing slightly different but complementary functions. This simpler plesiomorphic, “serial” head configuration has likely provided habeliidans with a proportionally much stronger raw crushing power—analogous to an insect, myriapod or malacostracan being equipped with four additional mandibles, albeit of different sizes. It is not known if these appendages were able to work in concert, creating a single movement of closure, or in series, as is the general condition for arthropod appendages; however, even if habeliid gnathobases were closing with a slight offset, the resulting movement still represented strength-wise the action of several consecutive mandibles.

Adding to the resemblance with mandibulates is the fact that the cephalic exopods were differentiated into antennule-like appendages, presenting a long and slender morphology, bearing stiff setae at the podomere junctions (Fig. 2c), and projecting at the front of the animal. Although external resemblance needs not always imply similar functions, this very peculiar form of exopods, in association with spinose endopods interpreted as having a raptorial role, leads to think that they were most likely used complementarily to sense the environment and prey items. We therefore regard them as sensory or tactile apparatuses (Figs. 3, 4, 6 and Additional file 8).

Whether this spectacular specialization was an autapomorphy of habeliidans or a plesiomorphic condition of chelicerates is not known, but similarities in the arrangement of exopodial branches in *Offacolus* and *Dibasterium* suggest that the first chelicerates may indeed have co-opted exopods as sensory/tactile features as a way to compensate for the lack of dedicated antennular appendage, locked by newly integrated developmental pathways. The later radiation of the group formed predatory niches discarding sensory/tactile apparatuses anteriorly in favour of median eyes, but a tactile function was to reappear in the arachnid pedipalps, and sensorial abilities through the modification of walking legs (endopods) in whip scorpions, whip spiders and many harvestmen.

The comparative picture is completed by the additional pair of appendages integrated to the head tagma—the “intermediary” appendage of *Habelia*, morphologically similar to those of the “mesosoma,” and the first maxilliped of mandibulates such as centipedes or isopods (Fig. 7). While it is argued here that this pair of appendages in habeliidans is plesiomorphic for Chelicerata, the integration of the first maxilliped into the head appears on the contrary highly convergent across mandibulates. Functionally, integrated maxillipeds represent an additional pair of limbs aiding in food manipulation, and this is also what we infer for the seventh cephalic pair in *Habelia* (and likely *Sanctacaris*). Given the difference in morphology between the latter relatively long appendages and the anterior enditic endopods, such a posterior pair would have helped sensing and maintaining the food in place during the action of the gnathobases (which is similar to the role of labial palps in many insects).

The size differentiation between cephalic gnathobases, differential length of teeth and development of spinose endopods as well as sensory/tactile rami point to a combination of active predatory, prey-grasping lifestyle with a type of food processing concentrated in the mouth area. The abundance of trilobites and the rise of shelly metazoans in Cambrian seas have called for postulating an array of durophagous niches [90–93], of which the most prominent actors were likely artiopodans themselves [94–96] with their well-developed gnathobases. Some might have been more-or-less selective predators [95], others more scavengers [96], but these body plans have mostly stood—albeit controversially [93, 97]—in contrast to other possible large hard-shell feeders, anomalocaridids [98], which were swimming and equipped with grasping appendages. Although small in absolute size, the cephalic gnathobases of habeliidans seem to have specifically evolved as adaptations to durophagous niches. This is notably supported by the presence of trilobite fragments within the gut of the habeliidan *Wisangocaris barbarahardyae* [35].

## Conclusions

Habeliidans contribute to build a richer and more dynamic view of the benthic Cambrian faunas, in which small active predators also adapted to a diet based on hard and mineralized shells (Additional file 8). Arguably, strongly sclerotized mouthparts brought advantages other than the processing of thicker cuticles and shells (such as the possibility of developing new functions on other limb bases) and were not necessarily associated with such feeding habits (the small mandibles of *Tokummia* [9] and relatively delicate, specialized claws would rather point to soft-bodied prey items). Nonetheless, our general phylogenetic results and the new evidence provided by the study of *Habelia* indicate that the adaptation to durophagous niches may have broadly triggered the radiation of Artiopoda, Chelicerata and Mandibulata.

In its nature and extent, such morphological convergence between an early chelicerate and mandibulates has no other equivalent, and in that pertains the question of morphological variability and disparity among stem lineages. How much habeliidans would impact the early arthropod morphospace remains to be tested, but they represent an arguable departure from all related body plans, while sharing an ancestor with chelicerates and some morpho-functionality with mandibulates. In light of the evidence discussed above, namely sudden changes in head anatomy (from four- to seven-segmented) and appendage morphology (drastic modification of the basipod and “detachment” of exopods), as well as clues for possible upstream alterations in the development (parasegmental-like morphology of tergo-pleurae), *Habelia* seems to stem from a remarkable morphological variability in the common ancestor of chelicerates—as may pycnogonids and their challenging anatomies. Findings for very high evolutionary rates at the base of extant clades are consistent with this hypothesis [19]. This could support the idea that the Cambrian was marked by profound changes in gene regulatory networks [99], although the resulting macroevolutionary patterns may be more complex than an overall lower disparity for extant taxa [2, 100, 101].

The current palaeontological evidence suggests that habeliidans may not have survived beyond the Cambrian. If, as the convergence in the sclerotization of head appendages seems to suggest, they were competing with early mandibulates for small durophagous benthic prey items, they might not have been able to adapt to the transformation of those niches during the Ordovician, or may have been outcompeted by the ability of mandibulates to evolve various morphological specializations among head appendages. Owing to the view that poor developmental canalization could have been detrimental to the long-term fitness of stem taxa [102], the structural lability that habeliidans had inherited from the arachnomorph ancestor may have ultimately affected their likelihood of survival.

## Additional files

Additional file 1: Additional text including list of material, modifications of the phylogenetic matrix and comments on *Sanctacaris uncata* Briggs and Collins. (PDF 113 kb)

Additional file 2: Dataset file containing morphological data and code for phylogenetic analysis in MrBayes (Fig. 5), and segment data used for the variability graphs (Fig. 6). (TXT 44 kb)

Additional file 3: *Habelia optata* Walcott. (A-F) USNM 144908. (A) Full specimen, preserved latero-dorsally. Insets as indicated. (B) Close-up of spine-shaped pleura on posteriormost segment. (C) Close-up of thorax (mesosoma) and cephalon (prosoma). Insets as indicated. (D) Close-up of trunk pleurae. Arrowheads point to anterior margin of cuticular armature. (E) Close-up of head shield ornamentation, photographed in direct light. (F) Close-up of ornamental spines along the head shield margin. All pictures taken in cross-polarized light, unless otherwise indicated. See Methods for abbreviations. Scale bars: 5 mm (A); 1 mm (C, D, E); 0.5 mm (B, F). (JPEG 1911 kb)

Additional file 4: *Habelia optata* Walcott. (A-D) USNM 272169. (A) Full specimen, preserved in latero-dorsal aspect. Insets as indicated. (B) Close-up of distal portion of thoracic endopod, showing claw (podomere 1) and podomeres 2 and 3. (C) Close-up of distalmost portion of cephalic endopods, showing terminal claw and podomere 2 with well-developed endite. (D) View of entire thoracic endopod. (E-F) USNM 305091. (E) Full specimen, preserved in dorsal aspect. Arrowheads point to taphonomic breakage in telson. (F) View of entire thoracic endopod. All images using cross-polarizing light. Scale bars: 1 mm (A, D-F); 0.5 mm (B, C). (JPEG 2042 kb)

Additional file 5: *Habelia optata* Walcott. (A) ROMIP 64363, specimen preserved in latero-dorsal aspect; see also Fig. 2i. (B-D) ROMIP 64357; see also Figs. 1a, 2m. (B) Counterpart of (C), close-up of thorax (mesosoma) and cephalon (prosoma). (C) Full specimen before preparation, preserved in latero-dorsal aspect. Inset is (D). (D) Close-up of anterior region of prosoma, showing anterior reduced appendage and endpods 1–3, after preparation. (E-G) ROMIP 64364 (F, G counterpart of E); see also Fig. 2g–k. (E) Full specimen, preserved in dorsal aspect. (F) Close-up of prosoma. Inset is (G). (G) Close-up of labrum, hypostome and distal portion of cephalic endopods. All pictures taken in cross-polarized light. See Methods for abbreviations. Scale bars: 10 mm (E); 5 mm (A-C, F); 1 mm (D, G). (JPEG 1654 kb)

Additional file 6: *Habelia optata* Walcott. (A-D) USNM 139209; see also Figs. 1b, h, 2a. (A) Full specimen, preserved in latero-dorsal aspect. (B) Focus on tergite ornamentation using low angle plain light. (C) Close-up of cephalic and thoracic appendages. Note cheiromorph morphology of biramous mesosomal appendages. Inset is (D). (D) Close-up of eye. Arrowheads point to margin of ocular notch. (E-G) ROMIP 64368. (E) Full specimen, preserved in latero-dorsal aspect. Insets as indicated. (F) Close-up of posterior region. (G) Close-up of area beneath cephalic shield, showing exopod of intermediary appendage. Arrowhead points to margin of cephalic pleura. All pictures taken in cross-polarized light, unless otherwise indicated. See Methods for abbreviations. Scale bars: (A, B, E), 4 mm; (C), 2 mm; (D), 0.5 mm; (G, F), 1 mm. (JPEG 1989 kb)

Additional file 7: *Habelia optata* Walcott. (A, B) ROMIP 64370. (A) Full specimen preserved in dorsal aspect. Inset is (B). (B) Close-up of intestinal tract and wide stomach located within the cephalon. (C, D) ROMIP 64358, counterpart of Fig. 1c; see also Fig. 2d. (C) Full specimen, preserved in latero-dorsal aspect; composite image of both part and counterpart. Inset is (D). (D) Close-up of cephalic and thoracic appendages. Arrowheads point to overlapping bases of antennular exopod rami. We construe that the fifth spinose cephalic endopods was taphonomically displaced, as is suggested by the retracted position of the posteriormost gnathobases. (E, F) ROMIP 64359; see also Figs. 1d, e, 2n. (E) Twisted specimen preserved in latero-dorsal (trunk) and latero-ventral (head) aspect. Close-up of head and trunk. (F) Same as E, direct light. Arrowheads point to paired, serially repeated phosphatized structures of uncertain nature. Their dislocation from the intestine and atypical shape cast doubt on an interpretation as midgut glands. (G-I) ROMIP 64352; see also Fig. 2h. (G) Full specimen, preserved in latero-ventral aspect. Insets as indicated. (H) Close-up of metasomal exopods. (I) Close-up of prosoma. Left cephalic endopods are preserved stacked on top of each other next to their corresponding gnathobases. “Exopod” rami are preserved apart from the main appendage structure; their point of attachment is unclear. (J, K) ROMIP 64379, *H. optata*, possibly morph A, from the Tulip Beds (Mount Stephen). (J) Full specimen, preserved in latero-dorsal aspect. (K) Counterpart of J. All pictures taken in cross-polarized light, unless otherwise indicated. See Methods for abbreviations. Scale bars: 5 mm (A-C, G, I, J); 2 mm (E, F); 1 mm (D, H, K). (JPEG 1949 kb)

Additional file 8: Artistic illustration of *Habelia optata*. Courtesy of Joanna Liang © Royal Ontario Museum. (TIFF 9740 kb)

Additional file 9: *Sanctacaris uncata* Briggs and Collins, Holotype ROMIP 43502. Part (A) and partial counterpart (B) both discovered in 1983 - lower weathered portion of the counterpart never before published was discovered in 2007. (A) Full specimen, preserved in dorsal aspect. Insets as indicated. (B) Full specimen, counterpart. Inset is (C). (C) Close-up of anterior region of prosoma. Inset is (I). (D) Specimen photographed in direct light after coating in ammonium chloride sublimate. Arrowheads point to small dorsal carinae on trunk tergites. (E) Close-up of anterior trunk appendages on right side of body, possibly the corresponding exopods of (F) with setae not preserved. (F) Close-up of exopod of first and second trunk appendages. (G) Close-up of cephalic appendages posterior to raptorial “bundle,” showing paddle-like exopod interpreted as belonging to the intermediary appendage, and small appendage with distal setal brush of unclear identity. (H) Close-up on first cephalic endopod, showing five well-developed endites on inner margins of podomeres, and possibly an additional one proximally. (I) Close-up of frontalmost region, showing morphology of cephalic endopods 1–3. Endites indicated by asterisks. Ventral face of labrum revealing bipartite frontal morphology (demarcation pointed by arrow) with paired reflective spots. (J) Close-up of cephalic endopod claw. Arrowhead point at tooth on inner margin of main claw; arrow points at secondary claw behind main claw. All pictures taken in cross-polarized light, unless otherwise indicated. Additional abbreviations: ed., endite(s); ed*n*, endite *n*; iex, exopod of intermediary appendage; tex*n*, trunk exopod *n*. See Methods for remaining abbreviations. Scale bars: 10 mm (A, B); 5 mm (C, D); 1 mm (E-J). (JPEG 1526 kb)
