# Correction to: Mandibulate convergence in an armoured Cambrian stem chelicerate

**DOI:** 10.1186/s12862-018-1189-y

**Published:** 2018-05-29

**Authors:** Cédric Aria, Jean-Bernard Caron

**Affiliations:** 1 Department of Ecology and Evolutionary Biology, University of Toronto, Toronto, Canada; 2 Present address: State Key Laboratory of Palaeobiology and Stratigraphy, Nanjing Institute of Geology and Palaeontology, Chinese Academy of Sciences, Nanjing, China

## Correction

The original article [1] had 4 paragraphs which contained erroneous information. In this correction article the correct and incorrect information is shown.

- **Page 2**
  - **○ Incorrect:** In this study, we thoroughly reinvestigated the Burgess Shale euarthropod Habelia Walcott, 1912 based on Walcott’s original material and new specimens discovered by the Royal Ontario Museum. Habelia optata was initially regarded by Walcott as an “aglaspidid merostome,” which would hint at a chelicerate affinity [36], but this statement lacked much justification [37]. Simonetta [38] and Simonetta and Delle Cave [39] followed this view based mostly on overall aspect, while preferring to compare H. brevicauda, the new morphotype erected by Simonetta, to Leanchoilia [39]—a megacheiran. Importantly, early authors [37–41] recognized the presence of at least five pairs of head appendages, a condition that could have later related this animal to Sanctacaris—even if an interpretation of strictly five pairs and some other morphological details led to comparisons with crustaceans instead [40, 42]. In his revision of the genus, however, Whittington [43] rejected previous interpretations of a cephalon with five head appendages or more, leaving Habelia as a problematicum. Herafter, we reevaluate the significance of Habelia for the early evolution of chelicerates, as well as for the understanding of morphological convergence in the ecological context of the radiation of Cambrian euarthropods.
  - **○ Correct:** In this study, we thoroughly reinvestigated the Burgess Shale euarthropod Habelia optata Walcott, 1912 based on Walcott’s original material and new specimens discovered by the Royal Ontario Museum. H. optata was initially regarded by Walcott as an “aglaspidid merostome,” which would hint at a chelicerate affinity [36], but this statement lacked much justification [37]. Simonetta [38] and Simonetta and Delle Cave [39] followed this view based mostly on overall aspect, while preferring to compare H. brevicauda, the new species erected by Simonetta, to Leanchoilia [39]—a megacheiran. Importantly, early authors [37–41] recognized the presence of at least five pairs of head appendages, a condition that could have later related this animal to Sanctacaris—even if an interpretation of strictly five pairs and some other morphological details led to comparisons with crustaceans instead [40, 42]. In his revision of the species, however, Whittington [43] rejected previous interpretations of a cephalon with five head appendages or more, leaving H. optata as a problematicum. Herafter, we reevaluate the significance of H. optata [...]
- **Page 3**
  - **○ Incorrect**: Abbreviations used in figures…
  - **○ Correct**: Abbreviations used in figures **and additional files**…
- **Page 3**
  - **○ Incorrect**: Abbreviations used in figures: ag, anterior gnathobase; am, arthrodial membrane; an, anus; ap, anal pouch; att, endopod attachment on gnathobase; bas, basipod(s); ce, cephalic endopod(s); cen, cephalic endopod n; cel, left cephalic exopods; cpl, cephalic pleura; cx, cephalic exopod(s); cxn, cephalic exopod n; db, distal brush; dpex, distal part of exopod; ds, dorsal spine; dtp; distal telson piece; e, eye; en, endopod n; en, endopod; ex, exopod; das, dark stain;
  - **○ Correct**: Abbreviations used in figures: ag, anterior gnathobase; am, arthrodial membrane; an, anus; ap, anal pouch; att, endopod attachment on gnathobase; bas, basipod(s); ce, cephalic endopod(s); cen, cephalic endopod n; cel, left cephalic exopods; cpl, cephalic pleura; cx, cephalic exopod(s); cxn, cephalic exopod n; das, dark stain; db, distal brush; dpex, distal part of exopod; ds, dorsal spine; dtp; distal telson piece; e, eye; en, endopod n; en, endopod; ex, exopod;
- **Page 4**
  - **○ Incorrect**: “however, the presence of **eight post-cephalic** tergites and a pygidium would rather seem to indicate a relationship with Mollisonia”
  - **○ Correct:** “however, the presence of **seven post-cephalic tergites** and a pygidium would rather seem to indicate a relationship with Mollisonia”
