# Supplementary material for: Mandibulate convergence in an armoured Cambrian stem chelicerate
Source: BMC Evol Biol. 2017 Dec 21;17:261. doi: 10.1186/s12862-017-1088-7 (PMC5738823; doi:10.1186/s12862-017-1088-7)
Supplement: Supplementary file 1 — Additional text including list of material, modifications of the phylogenetic matrix and comments on Sanctacaris uncata Briggs and Collins. (PDF 113 kb) [file 12862_2017_1088_MOESM1_ESM.pdf]

ADDITIONAL TEXT

Supplementary methods

Material

1] Specimens from the Royal Ontario Museum, specimens organized by stratigraphic occurrence within the “thick” Stephen (Burgess) Shale Formation. Morphs A, B or ? and locality [Raymond Quarry (RQ), Walcott Quarry (WQ), Tulip Beds (TB)] indicated in brackets:

- + 9.9 m
  64369 [?, RQ]
- +120 cm
  64376 [A, WQ]
- + 60 cm
  64363 [B, WQ]
- - 40 cm
  64377 [A, WQ]
- - 120 cm
  64357 [B, WQ], 64358 [B, WQ], 64368 [B, WQ], 64373 [?, WQ]
- - 130 cm
  64359 [A, WQ], 64362 [B, WQ], 64367 [B, WQ], 64370 [?, WQ], 64371 [?, WQ], 64374 [?, WQ], 64375 [?, WQ]
- - 210 cm
  64378 [A, WQ], 64384 [?, WQ]
- - 235 cm
  64364 [B, WQ]
- - 320 cm
  64380 [?, WQ]
- - 360 cm
  64382 [?, WQ]
- - 380
  64383 [?, WQ]

- - 400

  61403 [A, WQ]
- Talus

  64360 [B, WQ], 64365 [B, WQ], 64366 [B, WQ], 64381 [?, WQ], 64379 [A?, TB]

2] Specimens from the National Museum of Natural History. All are from the Phyllopod bed within the Walcott Quarry. Morph A, B or ? in brackets: PAL 57693 [A], PAL 139209 [B], PAL 144907 [B], PAL 144908 [B], PAL 144909 [A], PAL 268927 [A], PAL 268931 [A], PAL 268938 [?], PAL 272169 [A], PAL 272180 [?], PAL 272202 [A?], PAL 305090 [A], PAL 305091 [?], PAL 305092 [B].

Modifications of the morphological matrix

We made some small modifications to the data matrix compared to its initial use (1). We improved the chelicerate section of the dataset by adding one character emphasized on this study and two characters put forward as critical in the early evolution of the clade (see a review in ref. (2)):

- Character 104: Partial dissociation of exopods from main limb branch in head tagma. 0. Absent, 1. Present (see Discussion).
- Character 150: Tergite of eighth somite (counting the ocular somite as the first) drastically reduced as a "microtergite." 0. Absent, 1. Present.
- Character 151: Post-cephalic appendages covered by sclerotic plates (opercula). 0. Absent, 1. Present.

Character numbers discussed in Table 1 are shifted accordingly with respect to the character list in ref. (1).

In addition, we modified three characters following new observations described in the present study (see Discussion). Character 32 (number of somites defining the head tagma) was set to 3 (=8 somites, or 7 pairs of appendages) for *Sanctacaris*, *Habelia* and *Offacolus* (the original state 3 tentatively coding 9 somites for the larva of *Pycnogonum* was removed). In character 38, a cephalothorax had been coded as present in those fossil and extant chelicerates integrating an additional pair of appendages into their prosoma, along with mandibulates also having trunk appendages integrated to their head shields. A "cephalothorax" with such definition was arguably plesiomorphic to both clades, allowing for the formation of their more inclusive cephalic tagmata compared to the likely initial euarthropod condition of five somites (1, 3). To be more informative,

coding a cephalothorax in their respective taxa should therefore be relative to the plesiomorphic condition of head anatomy in these clades. With our new coding of the number of somites in the head tagma in basal "panchelicerates" (char. 32), we restricted the cephalothoracic condition to mandibulates and therefore chelicerates were coded 0 for character 38. We also tentatively coded a cephalothorax as present for Euthycarcinoidea *sensu lato* (1) given the broad posterior extension of the head shield, encompassing up to five additional segments behind the "specialized post-antennal appendages." Character 113 was modified to exclusively code for the reduction of exopods on the fourth pair of appendages across all taxa (as the coding was partly redundant with char. 84 already dealing with the reduction of endopods).

Interestingly, we also had to remove the (Scorpiones, Araneae) clade from the set of backbone constraints we used, based on ref. (4). This enforced grouping was causing a broad inversion of polarity for the chelicerate clade, with habeliids being derived within a monophyletic "Xiphosura" and *Chasmataspis* and *Megalograptus* retrieved as derived arachnids, sister taxa to scorpions. Although we rejected this topological arrangement as a result of a clear polarization bias, it is worth noting that the phylogenetic position of Scorpiones has a strong impact of the overall topology of Chelicerata, and in particular that morphology seems to support a sister-group relationship of scorpions to other arachnids (as in ref. (5); but see ref. (6)).

Supplementary discussion

Sanctacaris uncata Briggs and Collins, 1988

Sanctacaris was previously regarded as at least a cheliceromorph (7, 8), but such statements were based on different interpretations of the head tagmatization than those proposed in the present paper. An interpretation of differentiated limbs in *Sanctacaris* as the exopods of the cephalic appendages was also used by Legg (8) as an argument for a chelicerate relationship, based on the segmented condition of similar exopodial branches in the Silurian *Offacolus* (9) and *Dibasterium* (10). We follow such view, especially given the "detached" condition of cephalic exopods in these taxa (see main text), but we disagree with other details of the interpretation. The spinose cephalic endopods 2 to 5, and likely endopod 1 as well, are composed of seven visible podomeres, including the distal claw complex, and the bases are not visible (we construe that the gnathobases equivalent to those of *Habelia* are confounded within the thick layers of fossilized cuticle at the front; Additional file 8). More importantly, appendages of three different types were labeled as the same

cephalic exopods on the holotype: a short, broad, and likely lobate appendage bearing lamellate setae; another short, but elongate appendage with a setal brush at its tip (considered to be the sixth appendage by Briggs and Collins (7)); and the long, segmented "antenna-like rami" overlapped by the endopods (Additional file 8). The short appendage bearing a setal brush is the only one that resembles the stenopodous exopods of *Offacolus* and *Dibasterium*, but its identity in *Sanctacaris* is not obvious. The lobate appendage, located at the back of the anterior "bundle," best corresponds to the similar rounded exopod associated with the seventh cephalic pair in *Habelia* (Additional file 8), and which would be large enough to jut out slightly anteriorly if compressed ventrally against the head shield. Accordingly, the elongate branch could be the endopod of the seventh appendage, but a setose termination would be odd in the context of arachnomorphs. This appendage could also be a differentiated fifth cephalic exopod, but its very posterior position makes this interpretation questionable. The identity of this appendage can therefore not be disambiguated at present.

Nonetheless the position of what we interpret as the cephalic exopods 4 and 5, well-behind their corresponding endopods, corroborates the interpretation of a morphological dissociation between the inner and outer rami of the biramous appendages. The holotype of *Sanctacaris* also displays a frontal protrusion identical to the labrum identified in *Habelia* (Additional file 8). Anteriorly, the protrusion is split into left and right elements, each bearing a small central reflective spot. This morphology extends the comparison with protocaridids, other hymenocarines and possibly some artiopods discussed in the main text, whose frontalmost labral organs are bilobed and/or display paired or more reflective remains (11). There seems therefore to be cumulative evidence for a common ancestry of that structure (independently from the accompanying sclerites) at the point of diversification between artiopods, mandibulates and chelicerates.

Supplementary references

1. Aria C & Caron J-B (2017) Burgess Shale fossils illustrate the origin of the mandibulate body plan. *Nature* 545:89-92.
2. Dunlop JA & Lamsdell JC (2016) Segmentation and tagmosis in Chelicerata. *Arthropod Structure & Development* 46(3):395-418.
3. Walossek D & Müller KJ (1998) Cambrian 'Orsten'-type arthropods and the phylogeny of Crustacea. *Arthropod relationships*, eds Fortey RR & Thomas R (Chapman & Hall, London), pp 139-153.
4. Regier JC, *et al.* (2010) Arthropod relationships revealed by phylogenomic analysis of nuclear protein-coding sequences. *Nature* 463(7284):1079-1098.

5. Selden PA, Lamsdell JC, & Qi L (2015) An unusual euchelicerate linking horseshoe crabs and eurypterids, from the Lower Devonian (Lochkovian) of Yunnan, China. *Zoologica Scripta* 44(6):645-652.
6. Garwood RJ & Dunlop J (2014) Three-dimensional reconstruction and the phylogeny of extinct chelicerate orders. *Peerj* 2.
7. Briggs DEG & Collins D (1988) A Middle Cambrian chelicerate from Mount Stephen, British Columbia. *Palaeontology* 31:779-798.
8. Legg DA (2014) *Sanctacaris uncata*: the oldest chelicerate (Arthropoda). *Naturwissenschaften* 101(12):1065-1073.
9. Sutton MD, Briggs DEG, Siveter DJ, & Orr PJ (2002) The arthropod *Offacolus kingi* (Chelicerata) from the Silurian of Herefordshire, England: Computer based morphological reconstructions and phylogenetic affinities. *Proceedings of the Royal Society of London - Biological Sciences* 269(1497):1195-1203.
10. Briggs DEG, Siveter DJ, Sutton MD, Garwood RJ, & Legg D (2012) Silurian horseshoe crab illuminates the evolution of arthropod limbs. *Proceedings of the National Academy of Sciences of the United States of America* 109(39):15702-15705.
11. Ortega-Hernandez J (2015) Homology of head sclerites in Burgess Shale euarthropods. *Current Biology* 25(12):1625-1631.
