# Supplementary figures and images for: Mandibulate convergence in an armoured Cambrian stem chelicerate
Source: BMC Evol Biol. 2017 Dec 21;17:261. doi: 10.1186/s12862-017-1088-7 (PMC5738823; doi:10.1186/s12862-017-1088-7)

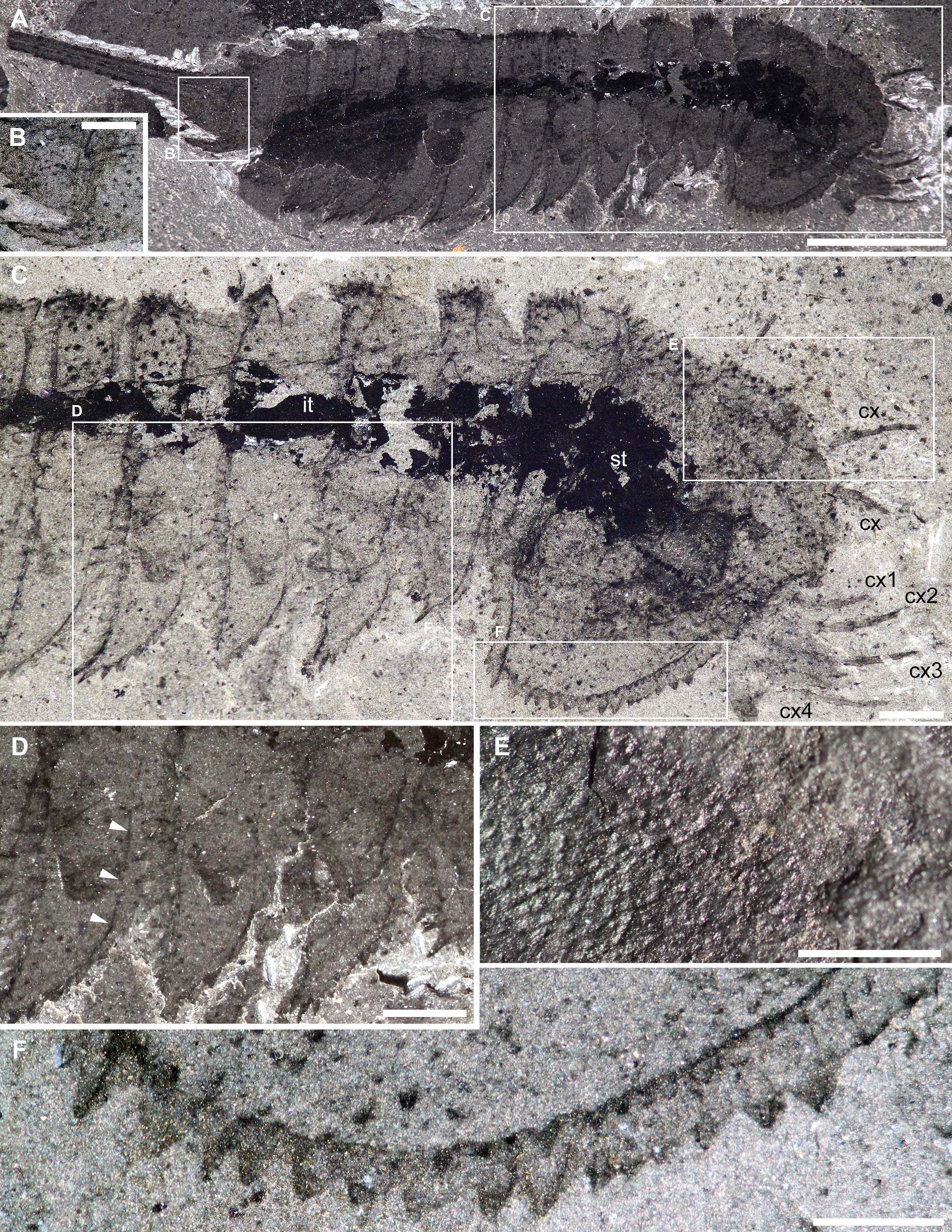

Supplement: Supplementary file 3 — Habelia optata Walcott. (A-F) USNM 144908. (A) Full specimen, preserved latero-dorsally. Insets as indicated. (B) Close-up of spine-shaped pleura on posteriormost segment. (C) Close-up of thorax (mesosoma) and cephalon (prosoma). Insets as indicated. (D) Close-up of trunk pleurae. Arrowheads point to anterior margin of cuticular armature. (E) Close-up of head shield ornamentation, photographed in direct light. (F) Close-up of ornamental spines along the head shield margin. All pictures taken in cross-polarized light, unless otherwise indicated. See Methods for abbreviations. Scale bars: 5 mm (A); 1 mm (C, D, E); 0.5 mm (B, F). (JPEG 1911 kb) [file 12862_2017_1088_MOESM3_ESM.jpg]

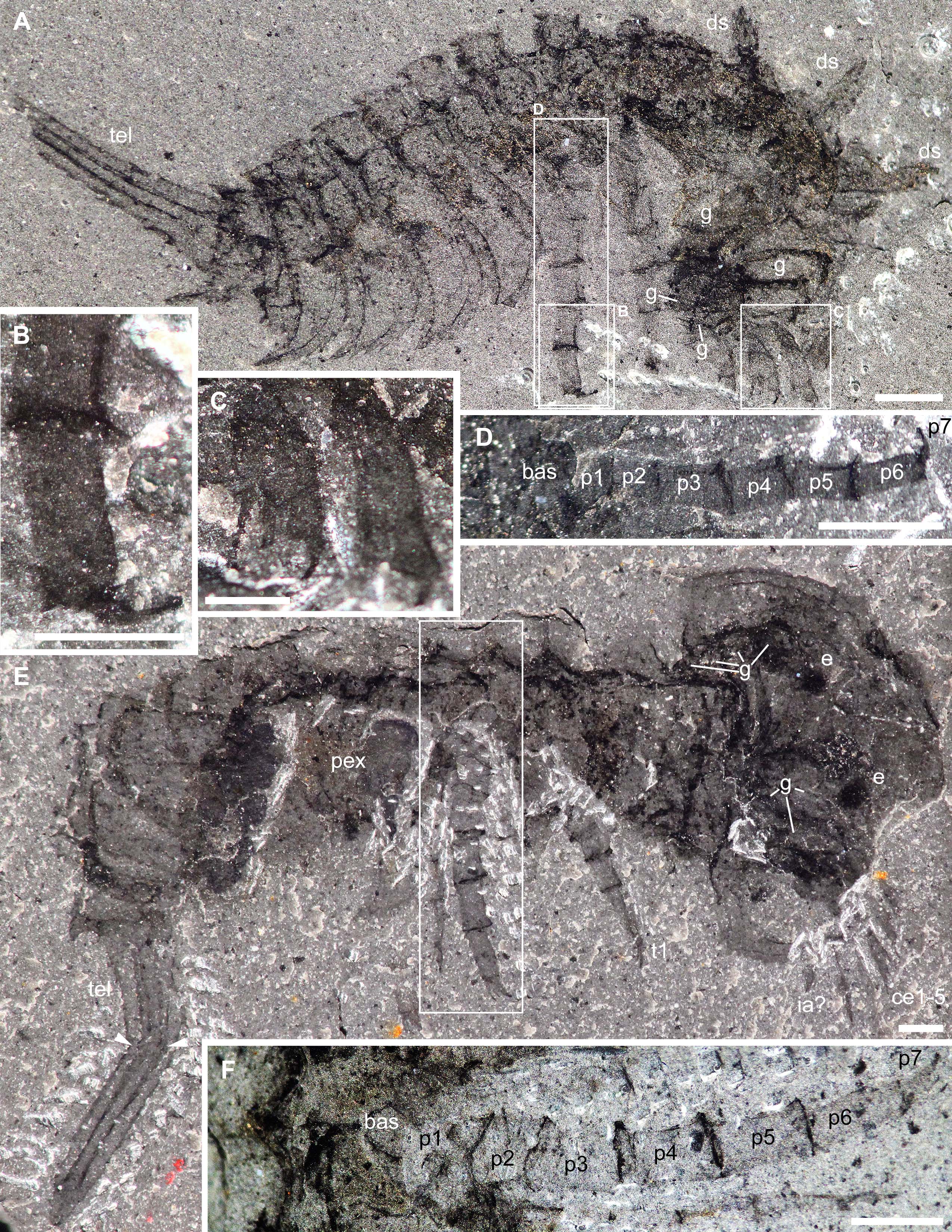

Supplement: Supplementary file 4 — Habelia optata Walcott. (A-D) USNM 272169. (A) Full specimen, preserved in latero-dorsal aspect. Insets as indicated. (B) Close-up of distal portion of thoracic endopod, showing claw (podomere 1) and podomeres 2 and 3. (C) Close-up of distalmost portion of cephalic endopods, showing terminal claw and podomere 2 with well-developed endite. (D) View of entire thoracic endopod. (E-F) USNM 305091. (E) Full specimen, preserved in dorsal aspect. Arrowheads point to taphonomic breakage in telson. (F) View of entire thoracic endopod. All images using cross-polarizing light. Scale bars: 1 mm (A, D-F); 0.5 mm (B, C). (JPEG 2042 kb) [file 12862_2017_1088_MOESM4_ESM.jpg]

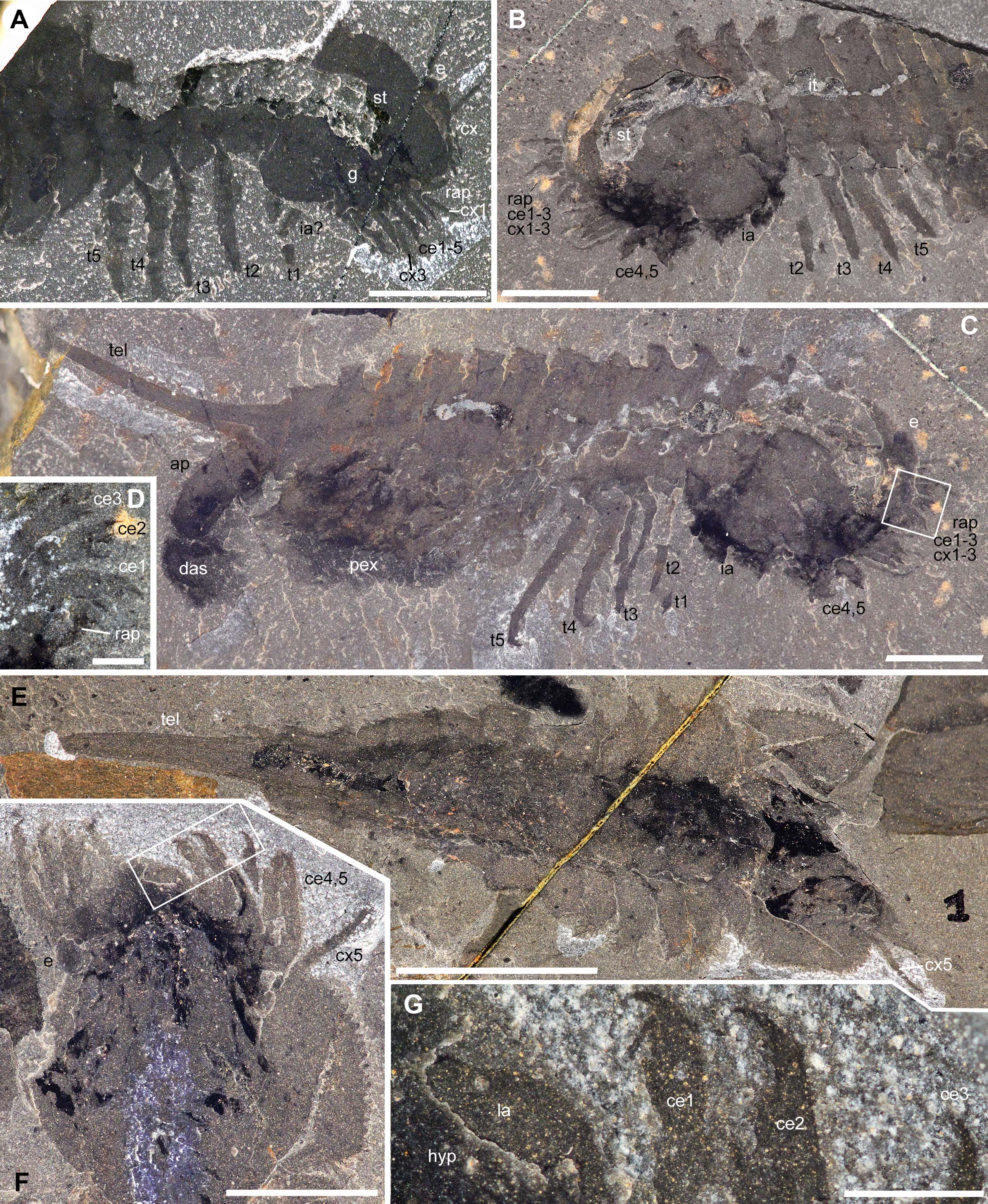

Supplement: Supplementary file 5 — Habelia optata Walcott. (A) ROMIP 64363, specimen preserved in latero-dorsal aspect; see also Fig. 2i. (B-D) ROMIP 64357; see also Figs. 1a, 2m. (B) Counterpart of (C), close-up of thorax (mesosoma) and cephalon (prosoma). (C) Full specimen before preparation, preserved in latero-dorsal aspect. Inset is (D). (D) Close-up of anterior region of prosoma, showing anterior reduced appendage and endpods 1–3, after preparation. (E-G) ROMIP 64364 (F, G counterpart of E); see also Fig. 2g–k. (E) Full specimen, preserved in dorsal aspect. (F) Close-up of prosoma. Inset is (G). (G) Close-up of labrum, hypostome and distal portion of cephalic endopods. All pictures taken in cross-polarized light. See Methods for abbreviations. Scale bars: 10 mm (E); 5 mm (A-C, F); 1 mm (D, G). (JPEG 1654 kb) [file 12862_2017_1088_MOESM5_ESM.jpg]

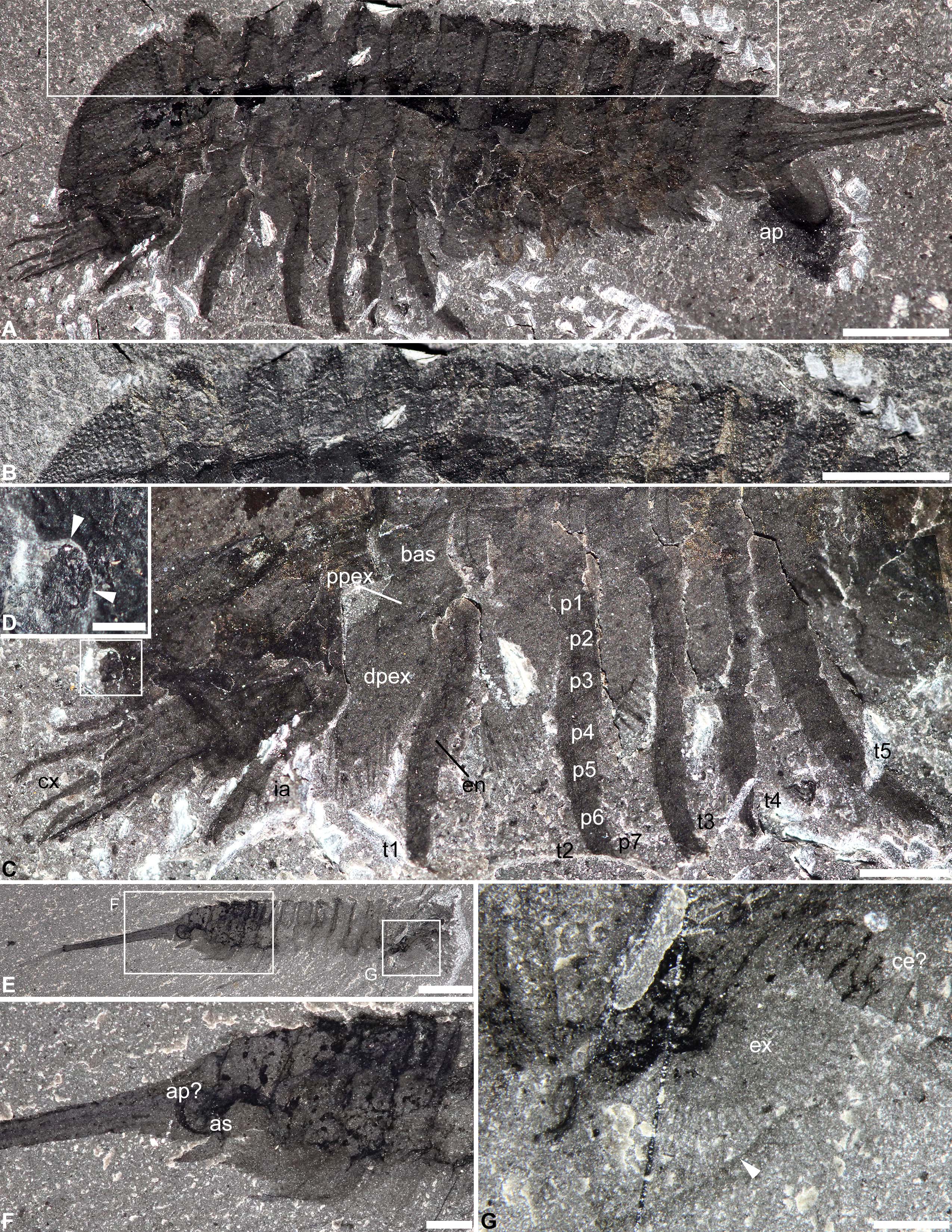

Supplement: Supplementary file 6 — Habelia optata Walcott. (A-D) USNM 139209; see also Figs. 1b, h, 2a. (A) Full specimen, preserved in latero-dorsal aspect. (B) Focus on tergite ornamentation using low angle plain light. (C) Close-up of cephalic and thoracic appendages. Note cheiromorph morphology of biramous mesosomal appendages. Inset is (D). (D) Close-up of eye. Arrowheads point to margin of ocular notch. (E-G) ROMIP 64368. (E) Full specimen, preserved in latero-dorsal aspect. Insets as indicated. (F) Close-up of posterior region. (G) Close-up of area beneath cephalic shield, showing exopod of intermediary appendage. Arrowhead points to margin of cephalic pleura. All pictures taken in cross-polarized light, unless otherwise indicated. See Methods for abbreviations. Scale bars: (A, B, E), 4 mm; (C), 2 mm; (D), 0.5 mm; (G, F), 1 mm. (JPEG 1989 kb) [file 12862_2017_1088_MOESM6_ESM.jpg]

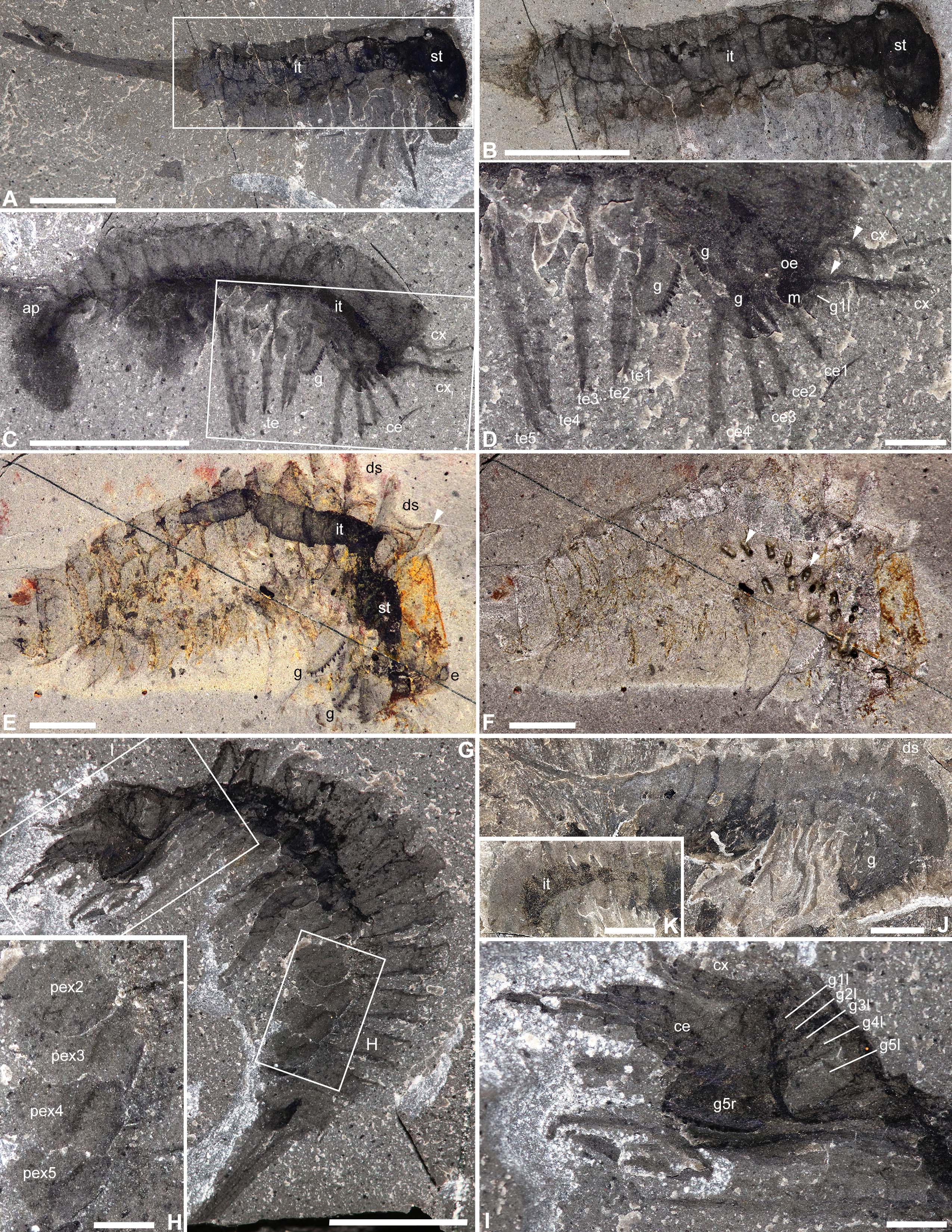

Supplement: Supplementary file 7 — Habelia optata Walcott. (A, B) ROMIP 64370. (A) Full specimen preserved in dorsal aspect. Inset is (B). (B) Close-up of intestinal tract and wide stomach located within the cephalon. (C, D) ROMIP 64358, counterpart of Fig. 1c; see also Fig. 2d. (C) Full specimen, preserved in latero-dorsal aspect; composite image of both part and counterpart. Inset is (D). (D) Close-up of cephalic and thoracic appendages. Arrowheads point to overlapping bases of antennular exopod rami. We construe that the fifth spinose cephalic endopods was taphonomically displaced, as is suggested by the retracted position of the posteriormost gnathobases. (E, F) ROMIP 64359; see also Figs. 1d, e, 2n. (E) Twisted specimen preserved in latero-dorsal (trunk) and latero-ventral (head) aspect. Close-up of head and trunk. (F) Same as E, direct light. Arrowheads point to paired, serially repeated phosphatized structures of uncertain nature. Their dislocation from the intestine and atypical shape cast doubt on an interpretation as midgut glands. (G-I) ROMIP 64352; see also Fig. 2h. (G) Full specimen, preserved in latero-ventral aspect. Insets as indicated. (H) Close-up of metasomal exopods. (I) Close-up of prosoma. Left cephalic endopods are preserved stacked on top of each other next to their corresponding gnathobases. “Exopod” rami are preserved apart from the main appendage structure; their point of attachment is unclear. (J, K) ROMIP 64379, H. optata, possibly morph A, from the Tulip Beds (Mount Stephen). (J) Full specimen, preserved in latero-dorsal aspect. (K) Counterpart of J. All pictures taken in cross-polarized light, unless otherwise indicated. See Methods for abbreviations. Scale bars: 5 mm (A-C, G, I, J); 2 mm (E, F); 1 mm (D, H, K). (JPEG 1949 kb) [file 12862_2017_1088_MOESM7_ESM.jpg]

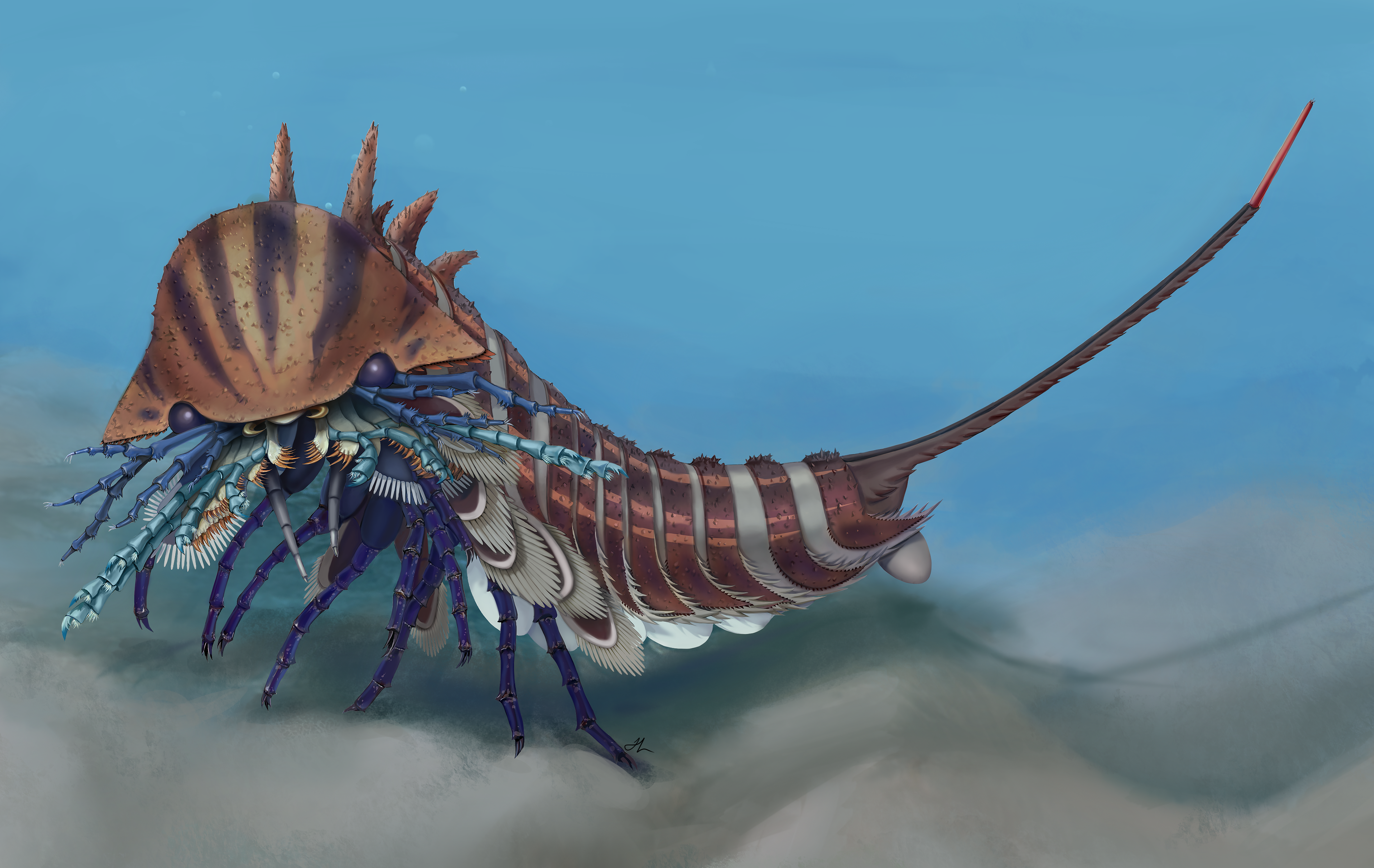

Supplement: Supplementary file 8 — Artistic illustration of Habelia optata. Courtesy of Joanna Liang © Royal Ontario Museum. (TIFF 9740 kb) [file 12862_2017_1088_MOESM8_ESM.tif]

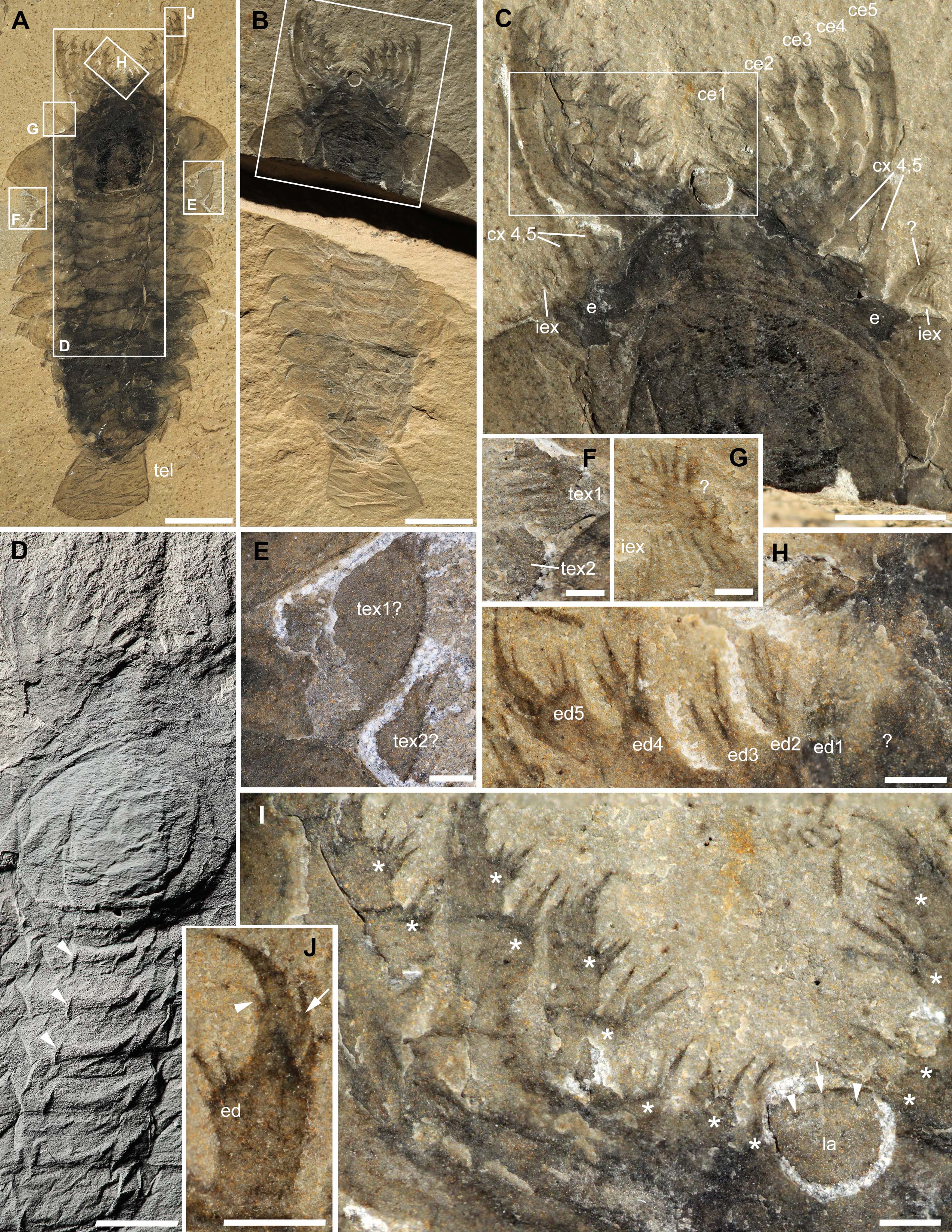

Supplement: Supplementary file 9 — Sanctacaris uncata Briggs and Collins, Holotype ROMIP 43502. Part (A) and partial counterpart (B) both discovered in 1983 - lower weathered portion of the counterpart never before published was discovered in 2007. (A) Full specimen, preserved in dorsal aspect. Insets as indicated. (B) Full specimen, counterpart. Inset is (C). (C) Close-up of anterior region of prosoma. Inset is (I). (D) Specimen photographed in direct light after coating in ammonium chloride sublimate. Arrowheads point to small dorsal carinae on trunk tergites. (E) Close-up of anterior trunk appendages on right side of body, possibly the corresponding exopods of (F) with setae not preserved. (F) Close-up of exopod of first and second trunk appendages. (G) Close-up of cephalic appendages posterior to raptorial “bundle,” showing paddle-like exopod interpreted as belonging to the intermediary appendage, and small appendage with distal setal brush of unclear identity. (H) Close-up on first cephalic endopod, showing five well-developed endites on inner margins of podomeres, and possibly an additional one proximally. (I) Close-up of frontalmost region, showing morphology of cephalic endopods 1–3. Endites indicated by asterisks. Ventral face of labrum revealing bipartite frontal morphology (demarcation pointed by arrow) with paired reflective spots. (J) Close-up of cephalic endopod claw. Arrowhead point at tooth on inner margin of main claw; arrow points at secondary claw behind main claw. All pictures taken in cross-polarized light, unless otherwise indicated. Additional abbreviations: ed., endite(s); edn, endite n; iex, exopod of intermediary appendage; texn, trunk exopod n. See Methods for remaining abbreviations. Scale bars: 10 mm (A, B); 5 mm (C, D); 1 mm (E-J). (JPEG 1526 kb) [file 12862_2017_1088_MOESM9_ESM.jpg]
